# The Lsm1-7/Pat1 complex binds to stress-activated mRNAs and modulates the response to hyperosmotic shock

**DOI:** 10.1371/journal.pgen.1007563

**Published:** 2018-07-30

**Authors:** Elena Garre, Vicent Pelechano, Manuel Sánchez del Pino, Paula Alepuz, Per Sunnerhagen

**Affiliations:** 1 Department of Chemistry and Molecular Biology, University of Gothenburg, Göteborg, Sweden; 2 SciLifeLab, Department of Microbiology, Tumor, and Cell Biology, Karolinska Institute, Stockholm, Sweden; 3 Departamento de Bioquímica y Biología Molecular, Universitat de València, Burjassot, Valencia, Spain; 4 ERI Biotecmed, Universitat de València, Burjassot, Valencia, Spain; Ohio State University, UNITED STATES

## Abstract

RNA-binding proteins (RBPs) establish the cellular fate of a transcript, but an understanding of these processes has been limited by a lack of identified specific interactions between RNA and protein molecules. Using MS2 RNA tagging, we have purified proteins associated with individual mRNA species induced by osmotic stress, *STL1* and *GPD1*. We found members of the Lsm1-7/Pat1 RBP complex to preferentially bind these mRNAs, relative to the non-stress induced mRNAs, *HYP2* and *ASH1*. To assess the functional importance, we mutated components of the Lsm1-7/Pat1 RBP complex and analyzed the impact on expression of osmostress gene products. We observed a defect in global translation inhibition under osmotic stress in *pat1* and *lsm1* mutants, which correlated with an abnormally high association of both non-stress and stress-induced mRNAs to translationally active polysomes. Additionally, for stress-induced proteins normally triggered only by moderate or high osmostress, in the mutants the protein levels rose high already at weak hyperosmosis. Analysis of ribosome passage on mRNAs through co-translational decay from the 5’ end (5P-Seq) showed increased ribosome accumulation in *lsm1* and *pat1* mutants upstream of the start codon. This effect was particularly strong for mRNAs induced under osmostress. Thus, our results indicate that, in addition to its role in degradation, the Lsm1-7/Pat1 complex acts as a selective translational repressor, having stronger effect over the translation initiation of heavily expressed mRNAs. Binding of the Lsm1-7/Pat1p complex to osmostress-induced mRNAs mitigates their translation, suppressing it in conditions of weak or no stress, and avoiding a hyperresponse when triggered.

## Author summary

When confronted with external physical or chemical stress, cells respond by increasing the mRNA output of a small number of genes required for stress survival, while shutting down the majority of other genes. Moreover, each mRNA is regulated under stress to either enhance or diminish its translation into proteins. The overall purpose is for the cell to optimize gene expression for survival and recovery during rapidly changing conditions. Much of this regulation is mediated by RNA-binding proteins. We have isolated proteins binding to specific mRNAs induced by stress, to investigate how they affect the stress response. We found members of one protein complex to be bound to stress-induced mRNAs. When mutants lacking these proteins were exposed to stress, ribosomes were more engaged with translating mRNAs than in the wild-type. In the mutants, it was also possible to trigger expression of stress proteins with only minimal stress levels. Tracing the passage of ribosomes over mRNAs, we saw that ribosomes accumulated around the start codon in the mutants. These findings indicate that the protein complex is required to moderate the stress response and prevent it from overreacting, which would be harmful for the cell.

## Introduction

The regulation of the gene expression is essential to all cells; therefore, the proper protein accumulation is controlled at multiple steps, including transcription and translation, as well as mRNA and protein transport and stability. During stable conditions, post-transcriptional control may explain some 20% of steady-state mRNA levels, however during rapidly changing conditions, post-transcriptional regulation is crucial for the initial responses [1]. As most investigations have focused on the general mechanisms acting on mRNA fate [2–4], little is known about the specific mechanisms for differential control of subgroups of genes. Stress represents an ideal scenario for studies of post-transcriptional regulation. Stress conditions force the cell to redirect its gene expression program. Until a cell has adapted to a sudden environmental change, it faces an acute energy shortage. Immediate survival prior to signaling-induced changes in gene expression depends on rapid post-translational events [5]. For medium-term adaptation and resumption of growth, the overall translation rate has to respond rapidly to changes in the environment, as protein synthesis represents a large fraction of the cell’s total energy expenditure. For rapid adaptations to preserve energy, post-transcriptional regulation acting on pre-existing mRNAs plays a major role by virtue of being faster than changes on the level of transcription initiation, and by intervening before the costly step of protein synthesis [6]. RBPs are fundamental for regulating transcript stability, localization, and translational efficiency, and thus sorting which mRNAs are to be expressed into proteins under specific conditions, ultimately contributing to stress survival and recovery. Thus, the mammalian RBP RBM3 protects neurons from cold shock [7], and glycine-rich RBPs in plants perform similar functions under cold and drought stress [8]. Under hypoxia in mammalian cells, an alternative translation initiation complex is formed at hypoxia-responsive mRNAs to enhance their translation, comprising eIF4E2 and RBM4 [9]. In yeast, the RBP Slf1 promotes translation of a few mRNAs during peroxide stress through interaction with the ribosome; *slf1* mutants are also hypersensitive to oxidative stress [10].

A hallmark of the acute stress response is a rapid and distinct global suppression of translation resulting from downregulation of ribosomal biogenesis factors and ribosomal proteins on multiple levels including initiation of transcription, transcript decay, and translational efficiency [11–14]. Pre-mRNAs encoding ribosomal proteins are subjected to the nonsense-mediated degradation pathway during osmotic stress [15]. In addition, transcript-specific regulation redirects the expression program to produce stress-protective proteins and reduce expression of proteins required for growth and proliferation. Stress induces a shift from cap-dependent to internal ribosome entry site dependent translation [16]. Oxidative stress causes increased translation of upstream ORFs (uORFs) and frameshifting events in mRNAs, potentially changing the proteome profile [17]. Specific RBPs are involved in promoting translation and inhibiting decay of mRNAs promoting stress survival [10,18–20]. Under severe stress, cytoplasmic stress granules (SGs) form, containing mRNAs to be silenced, ribosomal components and other RBPs. The majority of mRNAs are sorted to the stress-induced SGs and constitutive processing bodies (PBs), whereas the minority required for stress survival is kept out of these granules, to instead be selectively translated [21].

Combined, these observations raise the question how the fates of stress-activated mRNAs are determined under stress conditions. To reveal the mechanisms for this requires identification of the specific interactions taking place between individual RBPs and mRNA species. Such studies lag behind those of transcription factors and specific promoters, partly since RBP/RNA interactions are weaker than for DNA-binding proteins/DNA. Several global studies have identified RBPs associated with the total mRNA population in yeast and other organisms [2,3,22–27]. However, only very few studies have succeeded in isolating the proteins associated with a single mRNA species *in vivo*, *e*.*g*. [28–30].

In this study, we aim to answer this by isolating individual osmostress-activated mRNA species, quantitating the proteins associated *in vivo* with each of them, and analyzing how deletion of these proteins impact on the expression of stress-activated genes. By comparison with the proteome associated with individual, not stress-related, mRNA species, we identify specific proteins preferentially binding to osmostress-activated mRNAs, *STL1* and *GPD1*; notably members of the cytoplasmic Lsm1–7/Pat1 complex. The specific association between these proteins and *STL1* and *GPD1* mRNA was independently confirmed by quantitating stress and non-stress mRNAs bound to tagged proteins. We evaluated the impact of the Lsm1–7/Pat1 complex on the stress response by analyzing expression of osmostress-activated genes in *lsm1* and *pat1* mutants. We found those mRNAs to be more associated with polysomes than in the wild-type (wt). Moreover, the mutants fail to regulate the amount of the corresponding stress proteins under hyperosmotic shock. At low stress levels, the accumulation of those proteins is not triggered in the wt, but this occurs in the mutants. Finally, by mapping ribosome transit at single nucleotide resolution using 5P-Seq of mRNAs, we demonstrate increased ribosome accumulation in *pat1* and *lsm1* mutants in the near vicinity upstream of the start codon. This was particularly pronounced for specific subsets of mRNAs with functional enrichment in translation and mating components, and for mRNAs more highly transcribed and associated with ribosomes under osmostress. Together, these observations indicate that Pat1 and Lsm1 are important for dampening ribosome transit in the 5’-UTR, and thus translation initiation of osmostress mRNAs, in particular under low stress conditions. We conclude that our biochemical co-purification approach has successfully identified RBPs with a particular role in regulating post-transcriptional expression of stress-activated mRNAs.

## Results

### Isolation of RBPs associated with osmostress-induced mRNAs

**Fig 1:**
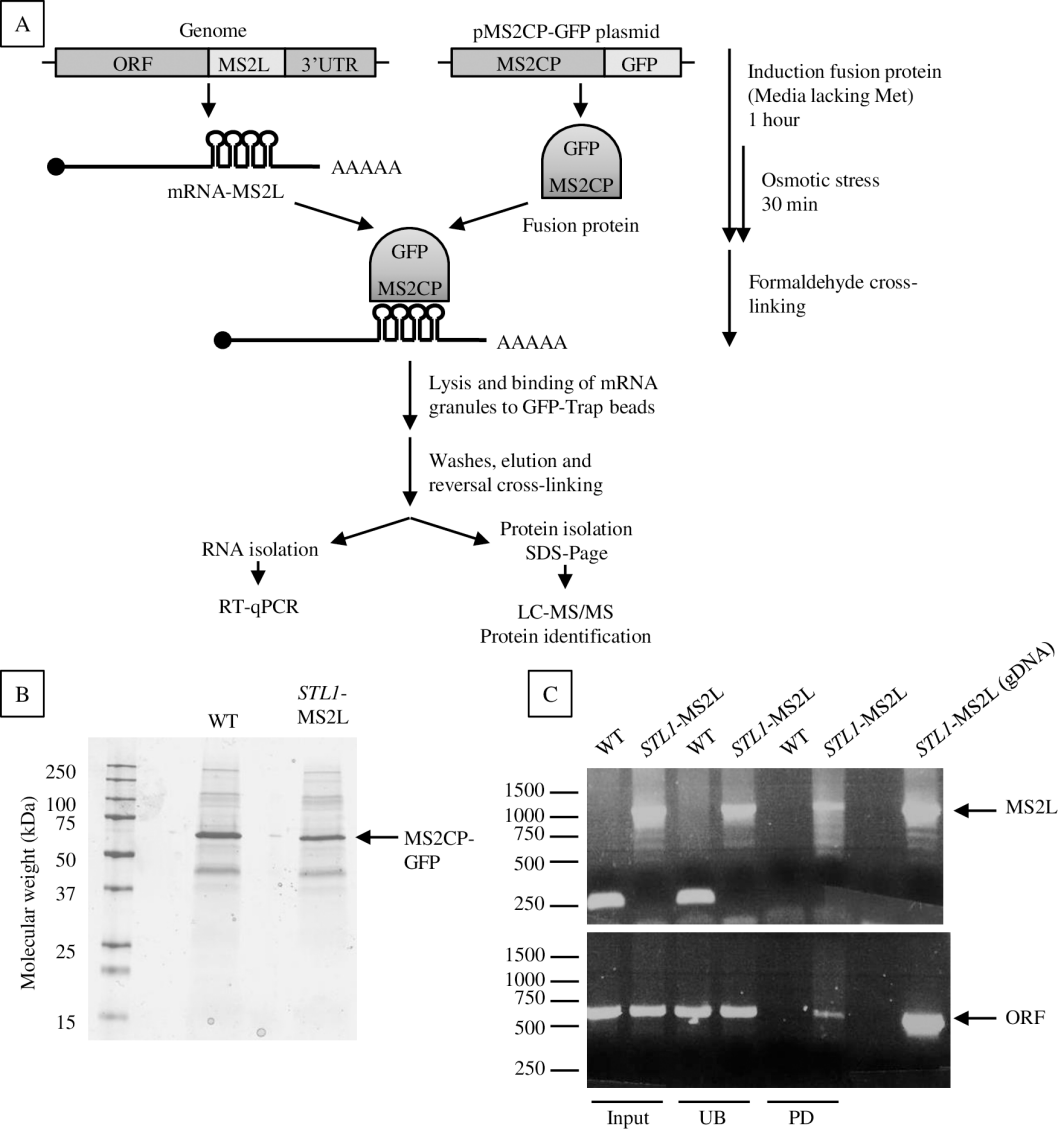
*In vivo* capture of RNPs associated with *GPD1* and *STL1* mRNAs under osmotic stress. A) Schematic outline of the immunoprecipitation–mass spectrometry procedure. After growing the cells to exponential phase, the expression of MS2-CP-GFP fusion protein was induced by methionine depletion and osmotic stress was applied by addition of KCl to 0.6 M. Proteins were then directly cross-linked to mRNAs *in vivo* by formaldehyde addition, after which RNPs-mRNA-MS2L interacting with the MS2-CP-GFP fusion protein were purified using GFP-Trap beads. After elution from the beads and cross-linking reversal, the sample was divided and RNA and proteins extracted separately. The proteins were separated by SDS-PAGE and composition was analyzed by LC-MS/MS. B, C) Example of RNP capture experiment using an *STL1-MS2L* mRNA containing strain. B) Protein separation by SDS-PAGE before digestion and mass spectrometry. C) *STL1-MS2L* mRNA detection by RT-PCR in input, unbound (UB) and pull-down (PD) samples. A pair of primers complementary to *STL1* and flanking the MS2 loops insertion region was used in upper panel. The MS2L integration was detected by a 1.0 kb amplification product. As a control of the integrity of the mRNA, a pair of primers within the ORF was used in the bottom panel.

Our goal was to identify RBPs involved in differential regulation of specific mRNAs under hyperosmotic stress. To isolate mRNA-specific ribonucleoprotein (RNP) complexes, we developed a method similar to those previously described [28,31] based on affinity purification of MS2 aptamer 3’-tagged mRNAs and subsequent protein composition analysis by LC-MS/MS, targeting directly the proteins interacting with individual osmo-mRNA species (Fig 1A, see Material and Methods). The MS2 loops were integrated into the genomic copies of *GPD1* and *STL1* genes (S1A Fig). *STL1* and *GPD1* are well-characterized for their high level of induction under moderate hyperosmotic stress in yeast (“osmo-mRNAs”) [32–34], under which condition they are also stabilized and actively translated [13,14,35]. They encode glycerol-3-dehydrogenase and a membrane-bound H^+^/glycerol symporter, respectively. Both are involved in intracellular accumulation of glycerol, the principal compatible osmolyte in yeast and essential for its survival under osmotic stress. As contrasting examples of osmostress non-responsive mRNAs, the *ASH1* and *HYP2* mRNAs were also tagged with MS2 loops. Under osmotic stress, *ASH1* and *HYP2* are not transcriptionally induced; these transcripts are instead destabilized and translationally inactivated [13,14,35]. The induction patterns and polysome profiles upon osmostress of these aptamer-tagged mRNAs were similar to their untagged full-length counterparts (S1 Fig).

As an additional test to verify the functionality of the tagged mRNAs, we analyzed the intracellular localization of those mRNAs by visualization of the MS2CP-GFP fusion protein bound to them (S2A Fig). In each case, a punctate pattern of the GFP fluorescence appeared when mRNA-MS2L and the fusion protein were expressed. It was previously shown that certain mRNAs are found in granules where they are actively translated [36]. To test the nature of the GFP-positive granules, cells were treated with cycloheximide (CHX) before incubation in media with or without 0.6 M KCl (S2A and S2B Fig). Such treatment prevents the formation of PBs and SGs, most likely by trapping mRNA on polysomes [36,37]. An increase in the number of mRNA-MS2L granules was observed after CHX treatment, significantly pronounced for *STL1*-MSL2, *GPD1*-MSL2 and *HYP2*-MSL2 mRNA granules when it was followed by osmotic stress. The observed increase in mRNA granules in CHX-treated cells indicates an accumulation of ribosome-associated mRNAs in granules. As expected if these granules represent actively translating mRNAs, analysis of co-localization of those mRNA-MS2L granules with the PB marker Dcp2-RFP (S2C Fig) showed a lower percentage of PB signal overlapping with the stress-induced mRNAs *STL1*-MS2L (11%) and *GPD1*-MS2L (20%) than with the repressed mRNA *HYP2*-MS2L (44%). To rule out any effect of tagged mRNAs on PB formation, we quantitated the number of PBs during osmostress in cells expressing MS2L-tagged mRNAs and MS2CP-GFP. None of the tagged mRNAs caused a PB increase over cells not expressing tagged mRNAs (S2D Fig). Altogether, these observations corroborate that most *STL1* and *GPD1* mRNA granules were distinct from PBs, in agreement with previous work showing that active translation can be found associated to granules.

Before proceeding with LC-MS/MS analysis, the RNP isolation was optimized using *ASH1* mRNA and its well-established interaction with She2p [28,38,39] (S3 Fig). Then, from three independent experiments with the four MS2L-tagged mRNA and the non-tagged reference strain under osmotic stress (0.6 M KCl, 30 min), RNPs were isolated, and the protein compositions were subsequently analyzed. A corresponding analysis of unstressed cells was not feasible as the levels of *STL1* and *GPD1* mRNAs under such conditions are orders of magnitude lower. The presence of the MS2-CP-GFP protein and the specific mRNA-MS2L transcript was verified in the input and pull-down samples (Fig 1B and 1C). Also, the enrichment in mRNA-MS2L after pull-down relative to the input sample was monitored by RT-qPCR, showing that only the respective mRNA used to isolate the RNPs was enriched (Table 1). These observations together indicated that the captured samples were indeed enriched in the mRNA-MS2L and its interacting proteins.

**Table 1:** Detection of the mRNAs in the isolated RNP fractions by qPCR.

	mRNAs		
Strain	*ASH1*	*GPD1*	*STL1*
*ASH1*-MS2L	3.79	0.04	0.04
*STL1*-MS2L	0.08	0.07	13.98
*GPD1*-MS2L	1.16	82.83	1.15

[(PD_mRNA-MS2L_/I_mRNA-MS2L_)/(PD_noMS2L_/I_noMS2_)]

We identified 202, 76, 93, and 24 proteins reproducibly enriched (> 2-fold higher than the untagged strain) binding the *GPD1*, *STL1*, *ASH1*, and *HYP2* mRNAs, respectively. To identify those proteins enriched preferentially with the osmo-mRNAs *GPD1* and *STL1*, we selected the proteins significantly detected in both the *GPD1-MS2L* and *STL1-MS2L* strains, and > 3-fold more abundant in these strains than in the untagged strain and the *ASH1-MS2L* and *HYP2-MS2L* strains. According to these criteria, 21 proteins were listed as enriched in both the *STL1* and *GPD1* mRNA preparations (Table 2). As was the purpose of the experiment, we identified several proteins previously shown to bind RNA, including 8 proteins interacting with mRNA and one binding tRNA. In addition, we found several proteins not previously identified as RBPs, but potentially part of the osmotic stress response, *e*.*g*. the enzyme Glo2 and the transcription factor Usv1 (see Discussion) and proteins involved in cell morphogenesis and plasma membrane processes (Htb1, Sac7 and Dig1).

**Table 2:** Proteins with enriched binding to the *STL1* and *GPD1* mRNAs identified by MS.

			Enrichment peptides				Enrichment TopAny			
Category	Gene	Function	*GPD1/ASH1*	*STL1/ASH1*	*GPD1/HYP2*	*STL1/HYP2*	*GPD1/ASH1*	*STL1/ASH1*	*GPD1/HYP2*	*STL1/HYP2*
mRNA binding	*LSM1*	Decay	1,50*^§^	2,11*^§^	1,38*	1,99*^§^	2,80	3,59	1,33	2,11
	*LSM2*	Splicing and decay	1,86*^§^	1,70*^§^	1,15*	0,99*	2,17	2,48	1,66	1,97
	*LSM4*	Splicing and decay	1,39*	1,47*	1,93*^§^	2,00*^§^	2,69*^§^	3,17*^§^	2,47*^§^	2,95*^§^
	*LSM5*	Splicing and decay	0,71	0,57	0,98	0,84	3,48*^§^	4,25*^§^	2,18*^§^	2,94*^§^
	*LSM6*	Splicing and decay	-	-	2,15	2,77	13,45*^§^	14,07*^§^	2,02*^§^	2,64*^§^
	*PAT1*	Decay	1,99*^§^	2,08*^§^	1,84*^§^	1,92*^§^	2,37*^§^	2,83*^§^	2,38*^§^	2,83*^§^
	*EDC3*	Decay	2,27	2,50*^§^	1,93	2,17	4,46*^§^	4,52*^§^	1,77*^§^	1,83*^§^
	*PAP1*	mRNA polyadenylation and transport	1,05	0,93	0,75	0,62	2,69*^§^	1,76*^§^	1,31*	0,38*
rRNA binding	*EMG1*	Ribosomal small subunit biogenesis	0,92	0,89	0,42	0,39	3,47*^§^	3,83*^§^	-0,64*	-0,28*
Other functions	*PHO8*	Nicotinamide nucleotide metabolic process	1,69*^§^	1,55*^§^	8,07*^§^	7,93*^§^	9,70*^§^	11,74*^§^	7,69*^§^	9,73*^§^
	*GLO2*	Methylglyoxal catabolic process	12,03*^§^	11,49*^§^	4,62*^§^	4,08*^§^	17,13*^§^	15,39*^§^	5,99*^§^	4,24*^§^
	*HBT1*	Cell morphogenesis	0,33	0,12	0,08	-0,13	2,54*^§^	4,58*^§^	2,62*^§^	4,66*^§^
	*SAC7*	Controls organization of the actin cytoskeleton	7,75*^§^	8,17*^§^	7,43*^§^	7,85*^§^	-	-	-	-
	*DIG1*	Negative regulation of invasive growth	3,74*^§^	1,42*	3,05*^§^	0,74*	5,53*^§^	3,24*^§^	10,24*^§^	7,95*^§^
	*YGR026W*	Uncharacterized	-0,33	0,37	-1,02	-0,32	4,55*^§^	4,24*^§^	-0,24*	-0,55*
	*SRP40*	Preribosome assembly or transport	0,05	0,33	0,32	0,59	2,35*^§^	2,47*^§^	0,59*	0,71*
	*YAP1802*	Endocytosis	6,86	7,37	6,54	7,05	9,59*^§^	11,06*^§^	7,58*^§^	9,05*^§^
	*USV1*	Putative transcription factor	0,67	-0,13	0,98	0,18	2,36*^§^	3,48*^§^	-1,65*	-0,53*
	*YBR225W*	Uncharacterized	0,32	0,17	0,16	0,00	2,98*^§^	2,11*^§^	2,29*^§^	1,42*
	*YPL260W*	Uncharacterized	2,52*^§^	1,46*	1,89*^§^	0,83*^§^	3,08*^§^	2,45*^§^	1,80*^§^	1,18*
	*SRC1*	Linked to TREX	3,25*^§^	1,77*^§^	2,97*^§^	1,49*	2,66	0,67	2,77	0,79

*statistically significant

^§^ log_2_ ratio >1.5 (3-fold change)

### Members of the Lsm1-7/Pat1 complex are recruited to *GPD1* and *STL1* mRNA under stress

Notably, six out of the nine RBPs identified as specifically enriched in *STL1* and *GPD1* RNPs under osmotic stress are components of the Lsm1-7/Pat1 complex. Two Pat1-Lsm complexes have been recently reported, Lsm1-7/Pat1 in the cytoplasm and Lsm2-8/Pat1 in the nucleus [40]. We detected the cytoplasm-specific component Lsm1, whereas the nucleus-specific Lsm8 is absent from our data (Table 2; S1 Table), indicating that the cytoplasmic Lsm1-7/Pat1 associates with *STL1* and *GPD1* mRNAs.

It has been extensively described that the Lsm1-7/Pat1 complex plays a critical role in mRNA decay via the 5’-3’ pathway, interacting with the oligoA tail at the 3’-end of transcripts, and through a protein bridge also with the 5’-cap [41,42]. Through this association to mRNAs targeted for decay, the complex promotes decapping via unknown mechanisms [43,44]. The same complex has also been implicated in translational control [45–48]. Unexpectedly, our data showed enriched association of components of this complex to *GPD1* and *STL1* mRNAs under osmotic stress, when these transcripts are induced, stabilized, and actively translated. To independently corroborate this enrichment, we performed a RNP pull-down assay under the same experimental conditions, but using GFP-tagged versions of some of the Lsm/Pat1 complex components [49] and analyzing the specific mRNA content by qPCR (Table 3). Under osmotic stress conditions, both *GPD1* and *STL1* mRNAs showed enriched (> 2-fold) binding compared with *ASH1* to the four Lsm proteins tested (Lsm1p, Lsm3p, Lsm4p and Lsm7p), and Pat1p. Using the same criteria, Pat1p was also preferentially captured by *GPD1* and *STL1* mRNAs when compared with *HYP2* mRNA.

**Table 3:** mRNA pull-down using GFP-tagged proteins after 30 min under osmotic stress (0.6 M KCl). The mRNA enrichment to one another was calculated as the ratio (PD/I)mRNA1/(PD/I)mRNA2, where PD is the relative amount of the specified mRNA detected in the pull-down using an specific GFP-tagged protein and I (input) is the relative amount of that mRNA in the cell extract prior to the pull-down. The levels of specific mRNAs were quantified by qPCR and the unspecific RNA pulled down from an untagged strain (BY4741) was used to normalize. His2p and Clb2p were included as controls of proteins that do not bind those mRNAs.

	Ratio (PD/I)_mRNA1_/(PD/I)_mRNA2_			
GFP tagged protein	*GPD1/ASH1*	*GPD1/HYP2*	*STL1/ASH1*	*STL1/HYP2*
Pat1p	5.88*	3.30*	7.07*	3.97*
Lsm1p	5.40*	0.60*	7.86*	0.90*
Lsm3p	4.61	1.46	6.30	2.00
Lsm4p	4.87*	0.44*	5.12*	0.46*
Lsm7p	2.79	1.25	3.44	1.54
His2p	NM**	NM**	NM**	NM**
Clb2p	NM**	NM**	NM**	NM**

NM, non-measurable (mRNA2 was not detected in pull-down)

*Geometric mean of 3 independent experiments

**2 independent experiments

### Lsm1-7/Pat1 mutants have defects in global translation repression and ribosome distribution

We hypothesized that this association of the Lsm1-7/Pat1 complex to induced mRNAs could be related with two scenarios: one where highly induced and translated mRNAs recruit this complex to dampen the high induction signal and compensate for the transcriptional induction; or a second where this complex has an enhancing role in translation, as recently described for the decapping component Dhh1 [50]. To test those hypotheses, we first examined the phenotypes of *pat1* and *lsm1* mutants, with respect to steady-state mRNA levels, mRNA stability and global translational repression under osmotic stress. For all these experiments, native full-length mRNAs without the MS2L tag were studied.

**Fig 2:**
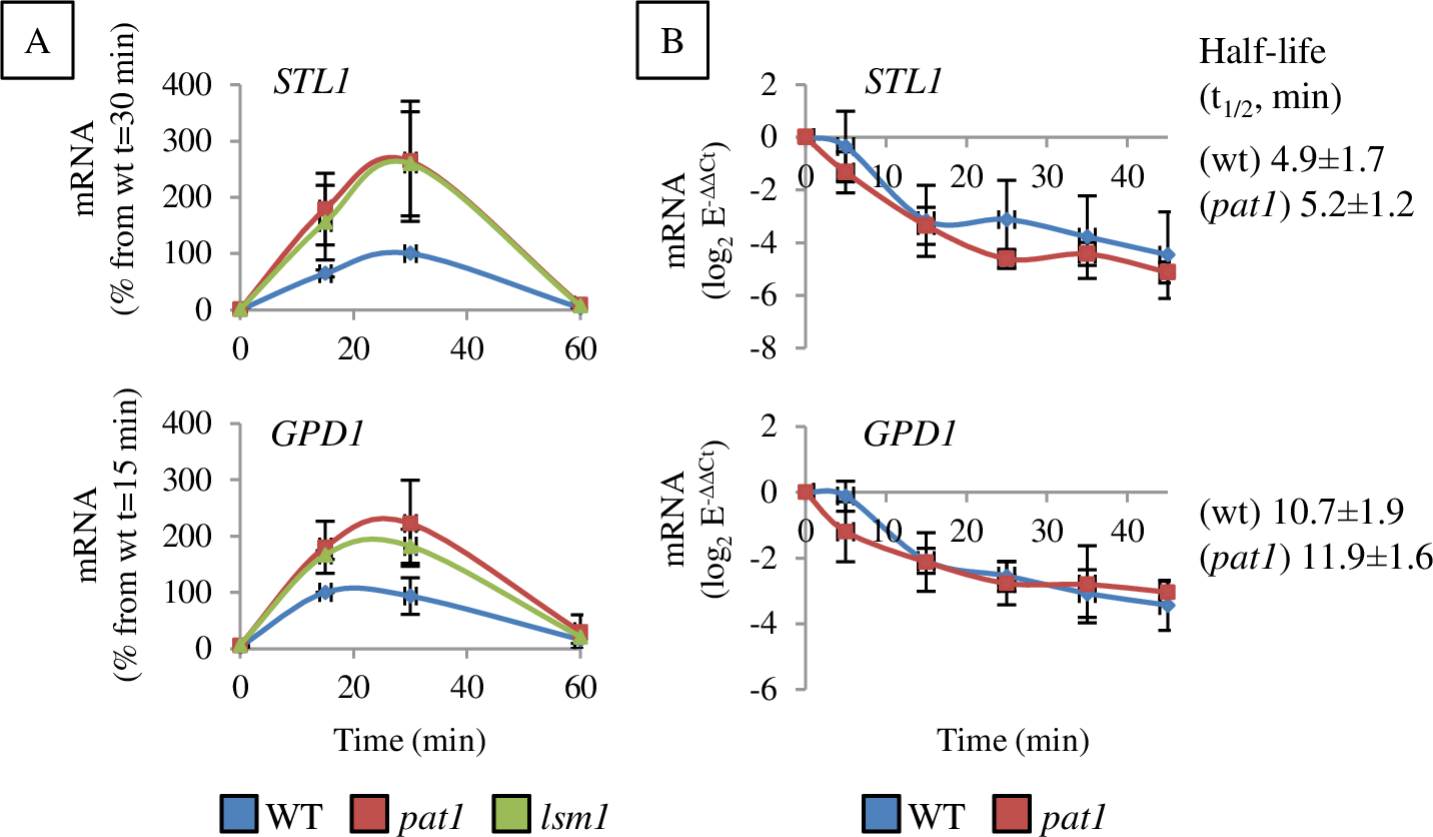
Deletion of Lsm1-7/Pat1 components affects mRNA levels of the osmo-mRNAs, *GPD1* and *STL1*, without major effects on stability. A) Steady-state levels of *STL1* and *GPD1* mRNAs in *lsm1* and *pat1* mutants under osmotic stress (0.6 M KCl). mRNA levels were normalized against the housekeeping gene *ACT1* and expressed relative to the highest value in wt strain. Average and standard error (SE) from three biological replicates are shown. B) Quantification of the mRNA decay rate after blocking transcription by 1,10-phenanthroline in wt and *pat1* strains under osmotic stress (30 min, 0.6 M KCl) and normalized against *ACT1* mRNA. Half-life ± SE from three independent experiments is indicated.

A time-course experiment was done in exponential growth cultures to measure the levels of *GPD1* and *STL1* mRNAs under osmotic stress in *pat1* and *lsm1* single mutants (Fig 2). Two to three times higher levels of *GPD1* and *STL1* mRNAs were observed in the *pat1* and *lsm1* mutants during stress than in wt (Fig 2A), although the accumulation kinetics of both mRNAs were similar between wt and mutants, reaching maximum levels 15 – 30 min of stress exposure. To evaluate if the observed mRNA accumulation in the *pat1* and *lsm1* mutants was consequence of their role in decapping, mRNA decay rates were analyzed after 30 min of osmotic stress and transcriptional shut-off by addition of 1,10-phenantroline (Fig 2B). No differences in decay rates for *STL1* and *GPD1* mRNAs between wt and *pat1* mutants were observed however.

**Fig 3:**
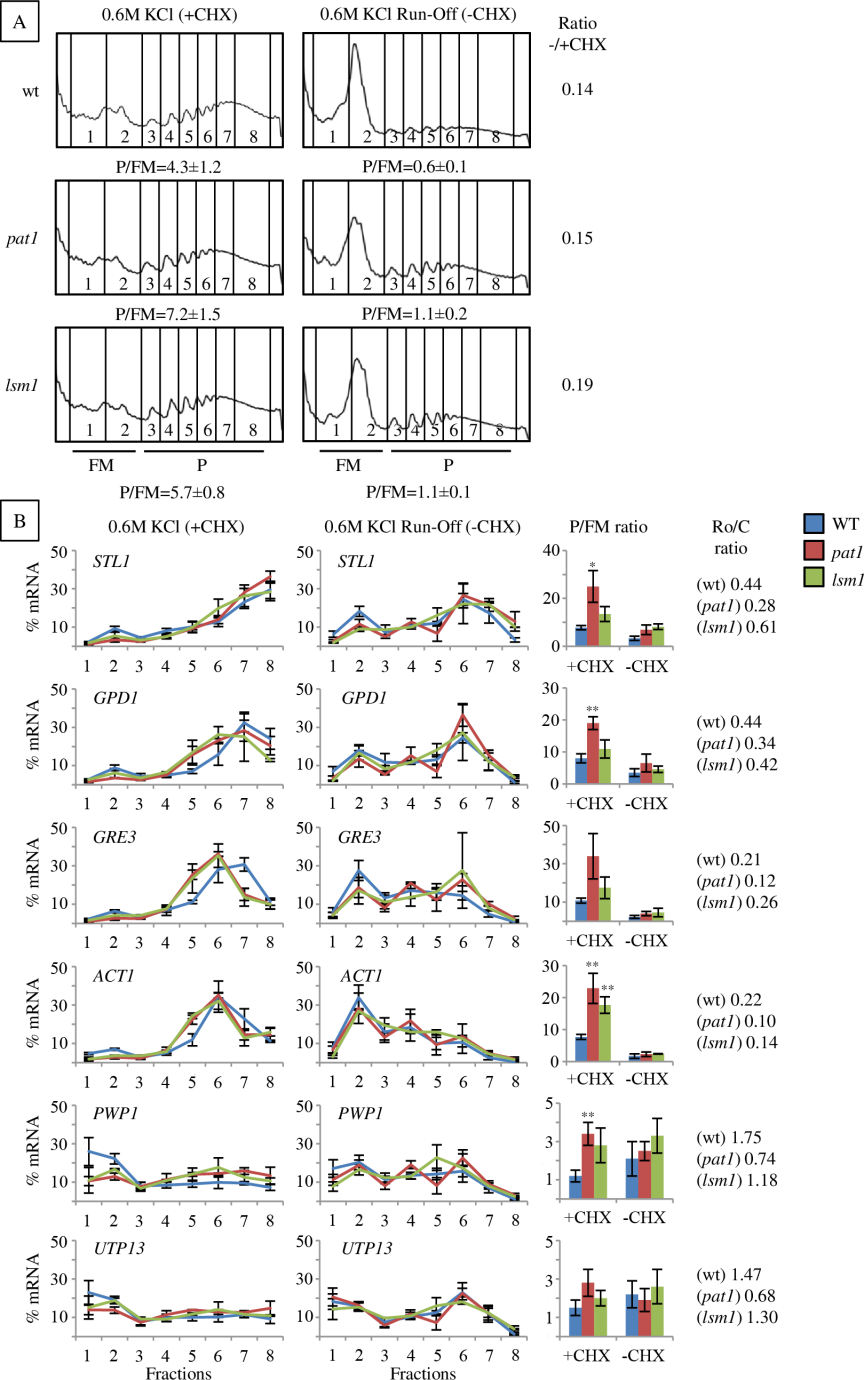
mRNAs show abnormally high polysome association in *pat1* and *lsm1* mutants under osmotic stress but the ribosomes can run off from them. A) Polysome profiles and B) specific mRNA association to them after 30 min of osmotic stress (0.6 M KCl) in the presence or absence (run-off) of CHX. P/FM, the ratio between polysomal RNA and sub-polysomal RNA (free plus monosomal RNA) is depicted below each profile in A) or in bar graphs in B). Average and standard error (SE) from three biological replicates are shown. In B, the RNA was prepared from individual fractions across polysome gradients as described in Material and Methods, and analyzed by qRT-PCR. The ratio P/FM run-off:P/FM control for each strain is indicated (Ratio -/+CHX). Statistical analyses were performed using Student’s t-test of comparisons between wt and mutants (*p<0.1, **p<0.05).

Next, we aimed to evaluate the impact of Pat1 and Lsm1 depletion on the regulation of translation under osmotic stress. For this, we first analyzed global translation profiles by separating polyribosomes (polysomes) by high-speed sedimentation centrifugation after 30 min of treatment with 0.6 M KCl (Fig 3, S4 and S5 Figs). Consistent with earlier results [20], after 30 min the cells had already recovered from the transient inhibition of the global translation (Fig 3A left panels). In *pat1* and *lsm1* mutants, the polysome proportion (P/FM ratio) was slightly higher than in wt cells. We verified that a proper global translation inhibition did occur in the wt strain at short times (6 and 15 min) when osmotic stress was added (S5A Fig); and also, as previously described for starved cells [47], that in the *pat1* mutant the ability to undergo translational repression under osmotic stress was impaired (S5A Fig).

To further analyze the impact of *pat1* and *lsm1* mutations in translation during stress, we investigated the association of specific transcripts with ribosomes (Fig 3B, left panels). In mRNAs associated with heavy polyribosomes in wt after 30 min of osmotic stress such as *STL1*, *GPD1*, *GRE3* and *ACT1*, we observed there was a reduction in the proportion of these mRNAs associated to non-polysomal fractions (1–2) in the *pat1* and *lsm1* mutants, turning out in a high P/FM ratio (Fig 3B, bar graphs). Similarly, the translationally repressed mRNAs *PWP1* and *UTP13*, abundant in non-polysomal fractions in wt, and the moderately repressed *HYP2*, were also more associated to polysomal fractions (3–8) in both mutants (Fig 3B left panels, bar graphs, S4 Fig). To better understand how these mutations affect the flux of those mRNAs on or off polyribosomes under osmotic stress, we analyzed their distribution at several short times after osmotic shock (S5B Fig). The stress-responsive *GPD1* and *STL1* mRNAs in *pat1* mutants accumulated in non-polysomal fractions (1–2) at a short time (6 min) after KCl addition similar to wt, and shifted to polyribosome fractions when the osmotic stress progressed. However, after 30 min was observed a markedly higher P/FM ratio in the *pat1* mutant (S5B Fig, bar graphs). By contrast, the stress-repressed *PWP1* and *UTP13* mRNAs were in high density fractions without stress and shifted to non-polysomal fractions under osmotic stress in wt, while remaining associated to polysomes in *pat1* mutants at 15 and 30 min. The *ACT1* mRNA, neither strongly upregulated nor repressed by osmostress, also showed higher accumulation to heavy fractions in the *pat1* mutant, and the *HYP2* mRNA, moderately repressed by osmostress, followed a similar pattern. For the non-induced mRNAs (*PWP1*, *UTP13*, *ACT1*, *HYP2*), the P/FM ratio was only moderately increased at 30 min. These results show that deletion of the *PAT1* gene resulted in increased progression of mRNAs to heavy fractions under osmotic stress for stress-induced transcripts, and in prolonged association to polysomes for stress-repressed transcripts.

Two potential explanations fit with our observations of higher mRNA polysomal association in *lsm1* and *pat1* mutants: 1) ribosomes stall during translation elongation and this could be due to impaired ribosome disassembly and recycling from the mRNAs; 2) an increased frequency of active ribosomes accessing the mRNAs. To discriminate between these options, we analyzed the ribosome run-off from polyribosomes in the absence of CHX in cells treated with 0.6 M KCl for 30 min (Fig 3A and 3B, right panels). To evaluate the effectiveness of run-off, we calculated the ratio P/FM [run-off] to P/FM [control] for each strain (Fig 3A). The proportion of run-off polysomes in *pat1* and *lsm1* mutants was not significantly different from the wt, indicating that most ribosomes are not irreversibly stalled. Consistent with this global analysis, the run-off of specific mRNAs from the polysomes into the non-translating fractions also showed only slight differences between *pat1* and *lsm1* mutants and the wt (Fig 3B, right panels and bar graphs). These observations are therefore most consistent with the second model, where the Lsm1-7/Pat1 complex may limit ribosome access to the mRNAs under osmotic stress.

### The *pat1* mutant fails to dampen the translation induction in stress-responsive mRNAs

**Fig 4:**
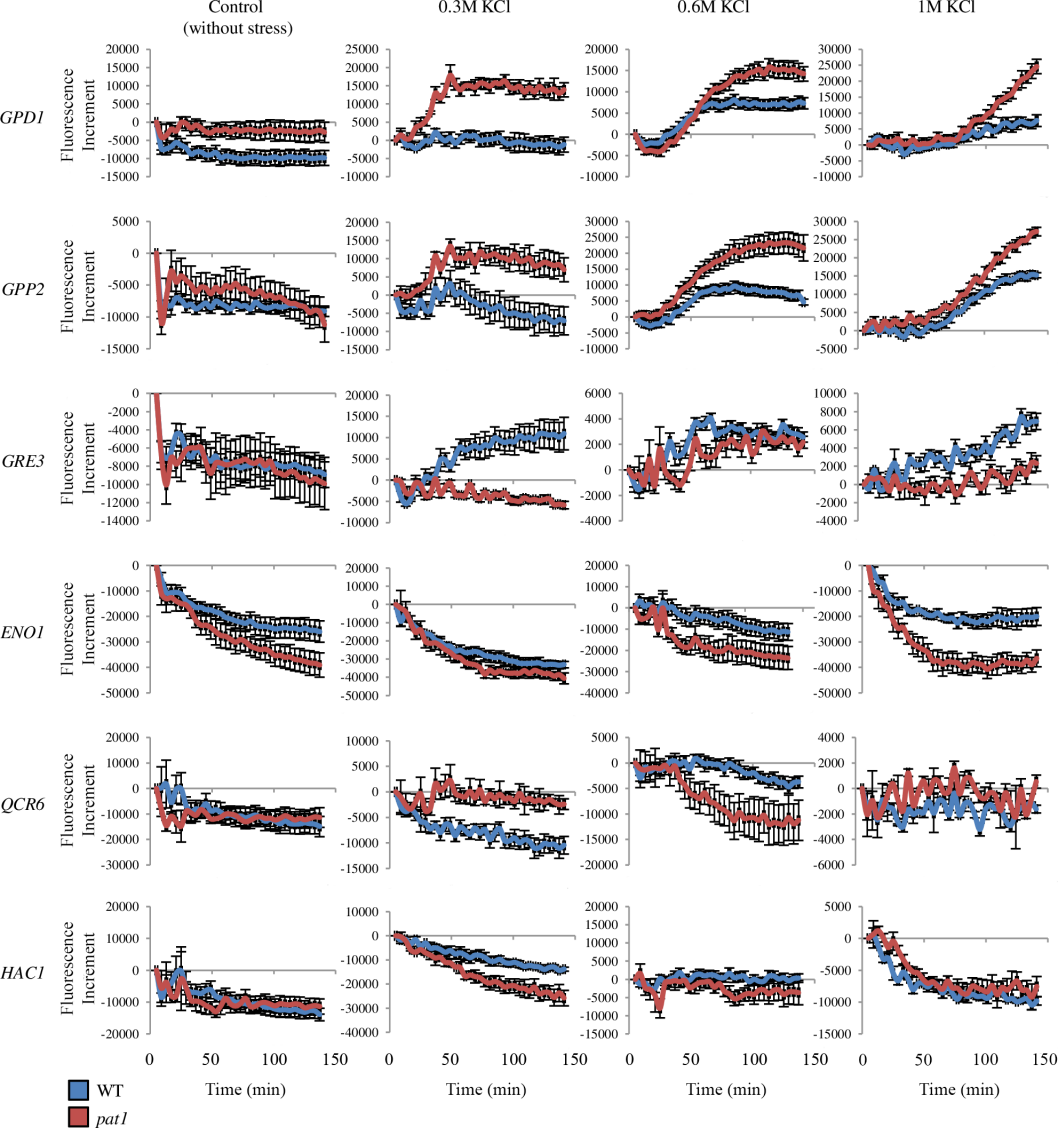
Deletion of *PAT1* dysregulates synthesis of highly induced proteins under osmotic stress. The accumulation of specific GFP-tagged proteins was monitored in *pat1* (red line) mutants and wt (blue line) in control conditions (without stress) and under osmotic stress by addition of KCl (mild, 0.3 M; medium, 0.6 M; high, 1 M). Protein accumulation was expressed as the increment of fluorescence units relative to 5 min after addition of KCl. Green fluorescence emission (520 nm) and OD_600_ was measured from the same well every 4 min for a period of 140 min in an Omega Polarstar fluorescence plate reader. To normalize the data, the OD_600_ was used to remove the effect of growth differences between strains, and *pat1* and wt with no GFP-tagged protein growing in parallel were used to subtract the background fluorescence. Average and SE from at least three biological replicates are shown.

The above results documented that the components of the Lsm1-7/Pat1 complex, Lsm1 and Pat1, regulate ribosome dynamics *in vivo*. To determine the output of this effect on protein levels and see if it was specific for subgroups of mRNAs under osmotic stress, we analyzed the accumulation of specific GFP-tagged proteins in a *pat1* mutant background. We selected a representative set of proteins, either strongly stress-induced or not induced, and for which the levels could be reliably monitored by GFP fluorescence. We measured the accumulation of the GFP-tagged proteins in the presence of KCl through a panel of different concentrations from low to medium severe stress (0.3, 0.6, and 1 M) (Fig 4).

Three different expression patterns were observed in the *pat1* mutant under osmotic stress. The first pattern, represented by Gpd1 and Gpp2, showed a striking increment in protein levels in this mutant under low to medium osmostress (0.3 M KCl). By contrast, no or low accumulation of those proteins was triggered in wt under the same conditions. Moreover, in higher salt concentrations (0.6 and 1 M KCl), when accumulation of those proteins was triggered in the wt, *pat1* mutants displayed 2–3 fold higher GFP signal. The second pattern was shown by Gre3, where a similar or even smaller GFP signal increment was detected in *pat1* mutants than in wt in presence of KCl. To estimate the contribution of the mRNA amount to the protein synthesis, we calculated the ratio between the areas under the curve of mRNA (S6 Fig) and protein (Fig 4) after shock with 0.6 M KCl (for protein, the area was calculated till the levels reached a plateau). The *pat1* mutant showed an increased production of 1.7 and 5.0 times more Gpd1p and Gpp2p per mRNA amount than wt, respectively (S2 Table). This indicates that the increment over wt levels of these proteins was too high to be explained by the increases of the corresponding mRNAs in the *pat1* mutant. The third pattern was seen for proteins not induced by the assayed osmostress conditions; Eno1p, Qcr6p and Hac1p. Here, the protein levels declined further in *pat1* mutants than in wt, and their changes under osmostress were uncorrelated with the changes in mRNA levels (Fig 4 and S6 Fig). Altogether, these data indicate that a deregulation of translation can be a significant cause of abnormal protein levels in *pat1* mutants.

These results are compatible with Pat1 being a translational repressor for highly induced osmo-mRNAs; however, it does not act homogeneously on all transcripts. Specifically, under osmotic stress, it dampens the protein levels from *GPD1* and *GPP2*, and prevents excessive accumulation of their encoded proteins. Still, other prerequisites for this effect probably exist as shown by the *GRE3* mRNA, which is also strongly induced at the transcript and protein levels, but where *PAT1* deletion has only a minor effect.

### Co-translational mRNA decay analysis indicates a ribosome overaccumulation upstream of the start codon in *pat1* and *lsm1* mutants

In order to further understand how the Lsm1-7/Pat1 complex affects the ribosome dynamics, we performed a genome-wide analysis of the ribosome protected regions in *pat1* and *lsm1* mutants using the recently developed 5P-Seq technique [51,52]. This approach offers an *in vivo* snapshot of ribosome footprints by sequencing 5’-phosphorylated mRNA co-translational degradation intermediates, and does not require the use of translation inhibitors or *in vitro* mRNA digestion. We performed three independent 5P-Seq experiments with each strain (wt, *pat1* and *lsm1* mutants) with or without osmotic stress (30 min, 0.6 M KCl).

**Fig 5:**
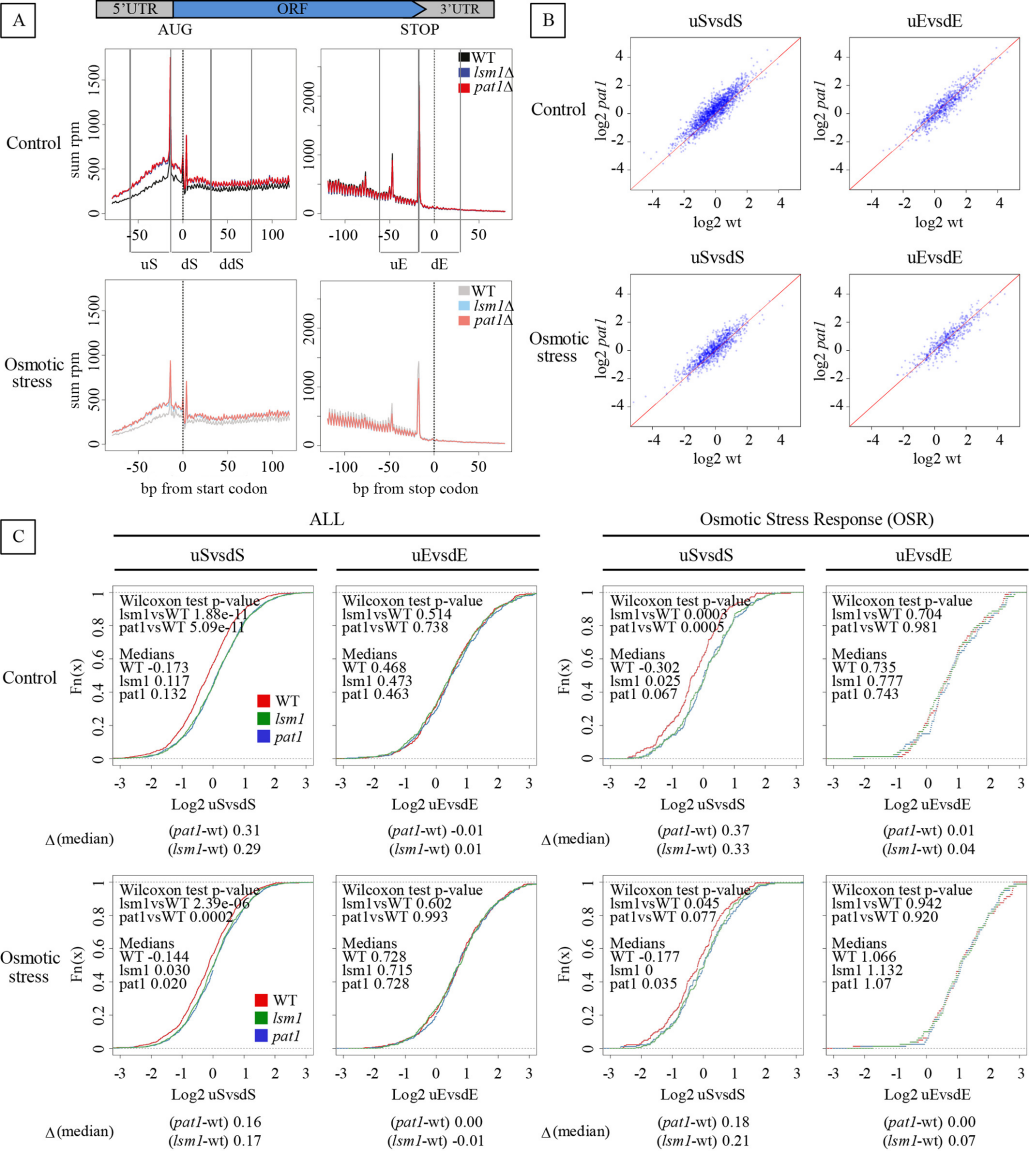
*pat1* or *lsm1* deletion yields overaccumulation of ribosomes in the 5’-UTR region of the mRNAs in both control and osmotic stress conditions. 5P-Seq was performed in *pat1* and *lsm1* mutants and wt strains before (control) and 30 min after addition of 0.6 M KCl (osmotic stress). A) Metagene analysis displaying the abundance of 5’P intermediates in reads per million (rpm) in relation to the ORF start (left panels) and stop codons (right panels), with (bottom panels) and without stress (upper panels). The graphics include the representation of the start region division in three windows of 45 nt each, upstream of the start codon (uS), downstream of the start codon including the ribosome paused at the start (dS) and, downstream of dS region (ddS); and the stop region in two windows, upstream of the stop codon including the ribosome paused at the stop (uE), and downstream of the stop (dE). B) Scatter plot representation and of log_2_ ratios between window areas around the start codon (uSvsdS) or the stop codon (uEvsdE) without (upper panels) and with osmotic stress (bottom panels). Only genes with at least 20 5P-Seq reads in the defined regions were considered for the analyses. Scatter plots display values for *pat1* mutant (Y axis) vs wt strains (X axis). Corresponding *lsm1* mutant plots are shown in S7B and S7C Fig) Cumulative representation of log_2_ ratios for all the transcripts sequenced (ALL) and for “osmotic-stress induced” gene subset defined in S6 Table (OSR). Increments of the cumulative distribution medians between wt and *pat1* and *lsm1* mutants, before and after 30 min of osmotic stress (0.6 M KCl), are displayed under each graph.

Alignment with respect to the start codon of the 5P-Seq reads, at the metagene level, showed a clearly increased ribosome accumulation around the translational start, almost identical for both *pat1* and *lsm1* mutants (Fig 5A, left panels). This ribosome accumulation was detected on both sides of the start codon (position -14 where ribosomes are paused at the P site during initiation [51,52]). A regular three-nucleotide pattern appeared at the start codon (Fig 5A), indicating the onset of active translation. This shift in periodicity was also clear from the analysis of the counts of protected molecules in the three frames (S7A Fig). As previously described, when the same analysis was repeated around the stop codon, the three-nucleotide pattern resulting from co-translational 5’ - 3’ mRNA degradation was observed, with a marked peak 17 nucleotides upstream of the stop. Here, however, no difference in ribosome protection was detected between wt and the *pat1* and *lsm1* mutants (Fig 5A, right panels). This difference being highest before the start codon, and absent in the 3’ region of the genes, indicates that cells depleted for Pat1 or Lsm1 present a general increased ribosome protection in the 5’ regions of the mRNAs, including the 5’-UTR. These differences cannot be explained by a hypothetical limited decapping in the mutants, as 5P-Seq measures only RNA molecules with a 5’ phosphate (*i*.*e*. after decapping). Therefore, independently of any potential capping differences, ribosomes accumulate in the 5’ regions of decapped mRNAs undergoing 5’-3’ decay.

To objectively measure the extent of this ribosome accumulation in the 5’ region, we calculated a loading ratio to compare different regions of the genes. We arbitrarily defined three 45 nucleotides (nt) regions around the start codon: upstream of the start codon (uS, between bases -60 and -15), downstream of the start codon (dS, -14 to 31 nt, including the ribosome paused at the start) and downstream of dS region (ddS, 32 to 77 nt within the ORF) (Fig 5A). To distinguish ribosome accumulation specifically in the 5’ region from overall differences in ribosome protection, we also defined two regions near the end region (-62 to -17 nt upstream and -18 to 45 nt downstream of the stop codon, uE and dE respectively). Two loading ratios were calculated covering the 5’ region (uSvsdS and uSvsddS) and one covering the 3’ end (uEvsdE) for each strain and each condition. A positive ratio for a gene thus indicates that there are more ribosome footprints towards the 5’ side of the analyzed region (*i*.*e*. ribosomes accumulate upstream). Consistent with the metagene analysis, the *pat1* and *lsm1* mutants present an increase in 5’-UTR ribosome protection also when analyzed at the single gene level (Fig 5B and 5C and S7B Fig). Comparing the 5’ regions, the distribution of the 5’ loading ratios indicating a global accumulation of ribosome footprints in the 5’-UTR in both the *pat1* and *lsm1* mutants increased notably relative to wt, showing more genes above the diagonal (Fig 5B left and mid panels); also observed by the shift towards positive values when plotting the cumulative distribution of gene-specific loading ratios (Fig 5C left and mid panels). The mutants showed more ribosome accumulation in uS than in dS, and these differences were more pronounced for the uS versus the ddS region (a region within the ORF). Similar differences, but at a lower degree, were also observed during osmotic stress (Fig 5A–5C, bottom panels). By contrast, no significant changes in 3’ loading ratios were observed confirming that the ribosome accumulation is specific of the 5’regions of the mRNAs (Fig 5B and 5C, right panels).

To determine if the 5’-UTR accumulation of ribosomes in *pat1* and *lsm1* mutants was general for the mRNA population, or if instead specific gene sets showed a different behavior, we performed a Gene Ontology (GO) analysis (http://babelomics.bioinfo.cipf.es/). To do so, we ranked the mRNAs according to a score based on their fold change of 5’-UTR ribosome accumulation in mutants relative to wt (calculated as log_2_ [uSvsdS_mutants_/uSvsdS_wt_]). This showed that different subsets of genes are clearly enriched among the mRNAs at both ends of the score distribution, both prior to and after stress (S5 Table). Prior to stress, the mRNAs with the highest score were enriched for genes involved in translation, nucleobase and amine metabolism, and transcription elongation. During stress, the highest scores were found with mRNAs related to signal transduction and response to stimulus and pheromone. The differences between the over-represented genes with and without stress were consistent with the two distinct biological environments: the enriched GO terms in actively growing cells were related to gene expression and protein synthesis, while under stress, to signal transduction and stimulus response.

As we found the Pat1 and Lsm1 proteins to be preferentially associated with the osmo-mRNAs, *GPD1* and *STL1*, we were interested to see if osmotic stress-induced genes had a higher 5’-UTR ribosome protection in *lsm1* and *pat1* mutants. We defined an “osmotic-stress induced” gene subset (Osmotic Stress Response; OSR) containing 544 transcripts, for which accumulation is induced under osmotic stress, using a compendium of several published mRNA expression datasets (details in S6 Table). The cumulative distribution of 5’-UTR ribosome protection for the OSR group, like the global graphs (Fig 5C), showed a higher overaccumulation of ribosomes around the 5’UTR in *lsm1* and *pat1* mutants compared to wt. This difference decreased after 30 min of osmotic stress. To evaluate if there were differences between the OSR group and the total population of mRNAs, we calculated the increment of cumulative distribution median between wt and mutants (Fig 5C). Interestingly, the OSR group showed higher increments that were statistically significant along the 5’UTR (uSvsdS) than the main group of analyzed transcripts under unstressed conditions, however not under stressed conditions (S4 Table). These observations indicate that the effect of Pat1 and Lsm1 on ribosome distribution along the 5’-UTR is more pronounced for transcripts encoding osmotic stress-induced proteins.

Together, the 5P-Seq results indicate that ribosomes may load more frequently onto the 5’-UTR of mRNAs in *lsm1* and *pat1* mutants, resulting in overprotection of this region; this effect is strongest in unstressed cells. In view of the enhanced production of Gpd1 and Gpp2 protein in the mutants under weak stress (Fig 4), this is indicative of increased recruitment of 40S subunits and translation initiation. Lsm1 and Pat1 may thus limit ribosome access and translation of mRNAs, in particular for stress-responsive mRNAs before stress is imposed.

## Discussion

### Osmo-RNP complexes are enriched in proteins involved in the osmotic stress response

The set of RBPs associated with the total transcriptome has been assessed several times using different methodologies and from different organisms and cells [3,24–27,53]. Importantly, those studies identified the major mRNA-binding proteins forming the core of most mRNPs, and shed light on global post-transcriptional regulation and the importance of RBP/mRNA interactions for that. There are only few reported attempts to isolate the protein set associated with a single mRNA species *in vivo* [28–30,54]. Therefore, the regulation of individual mRNAs and subsets of transcripts by RBPs is poorly understood. Here, we isolated specific RNP complexes binding to individual osmo-mRNAs in *Saccharomyces cerevisiae* to understand the composition of these complexes and how they can regulate the fate of the associated mRNA. We modified the MS2 aptamer-tagged mRNAs method [28,31] and used it to identify 21 proteins with preferential binding to the *GPD1* and *STL1* osmo-mRNAs under osmotic stress (Table 2). The use of MS2 aptamer-tagged mRNA methods has been questioned, reasoning that the MS2-CP-GFP protein binding to the MS2-tagged mRNA might block the 5’-3’ degradation and thereby stabilize 3’mRNA fragments and bias the results [55,56]. On the other hand, numerous publications where the MS2-MCP system was used have shown that the transcripts detected by MS2-MCP are intact, and their copy number and localization similar to untagged endogenous mRNAs (reviewed in [57]). In this study, we verified that the mRNAs tagged with MS2 sequences displayed a behavior upon stress similar to the corresponding native mRNAs, both with respect to transcriptional induction and polysome profiles (S1 Fig). Moreover, they did not preferentially co-localize with PBs (S2 Fig), and the punctate pattern of *STL1*-*MS2L* mRNA was similar to the native mRNA using FISH [58]. Importantly, in our study all the ensuing functional assays, showing altered behavior of stress-activated mRNAs in *lsm1* and *pat1* mutants were performed with native, untagged, full-length mRNAs.

In the last decade, several studies have reported novel RBPs. Most of them are well-characterized enzymes, now proposed to have a “moonlighting” role as post-transcriptional regulators [4,24,59,60]. In our study we identified 12 proteins not previously described as RBPs, out of 21 preferentially binding *STL1* and *GPD1* mRNAs. Several hypotheses about why these proteins bind RNA and/or form part of RNPs are on the table. There are examples of enzymes that post-transcriptionally regulate specific target mRNAs, such as cytosolic aconitase/IRP1 and GAPDH [59,61]. It has also been proposed that the RNAs could regulate those proteins, by competing with substrates for enzyme binding sites, or as assembly scaffolds for alignment of enzymes in a biochemical pathway [24]. Moreover, they can be part of the spatiotemporal regulation of signaling molecules, as described for the sequestration of TORC1 in SGs during heat stress [62]. Even though the role of those newly identified RBPs remains unknown, most of them are clearly related to the osmotic stress response. An interesting example is the methylglyoxal-catabolism enzyme Glo2, shown here to bind *GPD1* and *STL1* mRNAs. Cross-regulation between the glycerol synthesis and methylglyoxal catabolism at multiple levels has already been demonstrated [63–65]. Our present findings suggest new levels of this regulation. Another candidate to be involved in posttranscriptional regulation would be the sodium stress-response transcription factor Usv1 [66]. Our results suggest that many proteins associate with mRNAs and possibly influence their fates, providing dense connections between different layers of cellular regulation.

### The Lsm1-7/Pat1 complex binds to stress-activated mRNAs and coordinates their translation under osmotic stress

An unexpected finding was the enrichment of several components of Lsm1-7/Pat1 complex binding *GPD1* and *STL1* mRNAs under osmotic stress conditions when these transcripts are stabilized and actively translated [14,20]. The Lsm1-7/Pat1 complex is considered a conserved player in 5’ to 3’ mRNA decay, linking deadenylation to decapping [41–43,67,68]. In yeast, this complex preferentially binds U-rich tracts near the 3’ end of oligoadenylated rather than polyadenylated mRNA [3,69,70]. Lsm1-7 is composed of seven Sm-like proteins forming a ring, and it is the C-terminal extension of Lsm1 that approaches the RNA-binding pockets of Lsm1-7 enhancing the RNA binding properties of the core [70,71]. Pat1 is a multifunctional protein interacting with several proteins involved in decapping, mRNA decay and translational repression, and the participation of the Lsm1-7/Pat1 complex in those processes has been reported in several studies [41,45,47,72].

Here we corroborate that Lsm1 and Pat1, and probably the entire Lsm1-7/Pat1 complex, are general translational repressors. Importantly, we show for the first time that this function is stronger for specific mRNA groups. This is particularly obvious in weak to intermediate osmotic stress (Fig 4), suggesting that the major role of Lsm1-7/Pat1 complex in translation regulation is to permit balanced responses to environmental changes. First, we show that a major role of Pat1 and Lsm1 binding *GPD1* and *STL1* mRNAs under osmotic stress is to inhibit their translation. We cannot dismiss additional effects on mRNA decay and transcription; but they may make minor contributions to the phenotype of *lsm1* and *pat1* mutants under the conditions investigated here. This is supported by several observations: (a) *pat1* mutants overaccumulate Gpd1 protein in response to osmotic stress (Fig 4); (b) we did not find other proteins related with mRNA decay interacting with the Lsm1-7/Pat1 complex significantly enriched together with *STL1* and *GPD1* mRNAs (S1 Table), such as Dcp1 and Dcp2 decapping proteins, the CCR4-NOT deadenylation complex components, the Dhh1 DEAD-box helicase, or the Xrn1 5’-3’ exonuclease [41,42,72], nor the RNA pol II components Rpb4 and Rpb7 [66]; (c) *STL1* and *GPD1* mRNA half-life did not change in *pat1* mutants under osmotic stress (Fig 2B); (d) a higher proportion of *GPD1* and *STL1* mRNAs is associated to polysomes in *pat1* and *lsm1* mutants under osmotic stress (Fig 3 and S5 Fig), and (e) 5P-seq analyses show increased presence of ribosomes on mRNAs around the translational start in *lsm1* and *pat1* mutants (Fig 5).

Second, we show that the inhibition of translation mediated by Pat1 and Lsm1 is not homogeneous and affects more strongly specific mRNA subsets depending on cell condition. This is supported by the following key findings: (a) the 5’-UTR ribosome overprotection showed by *pat1* and *lsm1* mutants affects specific mRNA sets (S5 Table), moreover the affected mRNA sets change between control and stress conditions; (b) under stress, *pat1* and *lsm1* mutants also showed higher levels of some of the osmo-induced proteins than wt, and also they are already induced at low hyperosmosis, when moderate or high osmostress are normally required for strong induction in a wild-type strain (Fig 4). This last finding is consistent with the high increment of 5’UTR ribosome overprotection in the *pat1* and *lsm1* mutants showed by the OSR set (Fig 5C and S7B Fig) in non-stress conditions, which diminished with the stress; suggesting that the most important effect of Lsm1 and Pat1 is to attenuate translation of these mRNAs under conditions of no or weak stress. It is noteworthy that other mRNAs such as *QCR6* and *HAC1* increase to higher levels during osmostress in *pat1* mutants than in wt (S6 Fig), but the corresponding proteins still do not accumulate more in the mutant (Fig 4).

A scenario such as osmotic stress shows the relevance of coordination of the different layers of gene regulation, to be able to respond in the right way to the changing environment and produce the adequate levels of proteins needed for the adaptation fast but also on the proper time. The cellular response to hyperosmotic shock has been shown to be graded with respect to the severity of osmostress; the higher the osmolarity, the higher the amplitude and the longer the duration of the increased expression of stress proteins. Counterintuitively, mild osmoshock produces a quicker expression response than severe osmoshock [34,73]. Imbalanced stress responses have several negative consequences for the cell viability and fitness. For example, the overexpression of *GPD1* with consequent high accumulation of glycerol does not increase cell osmotolerance, but rather impairs growth in some cases [74,75].

Here we show that Pat1 and Lsm1 proteins have a notable effect, preventing overaccumulation of the Gpd1 and Gpp2 proteins. These two enzymes form the short branch of glycolysis involved in glycerol synthesis and are under expression control by the HOG pathway under weak to moderate hyperosmosis. Moreover, the 5P-Seq data reveal that numerous mRNAs encoding components of the pheromone response pathway accumulate ribosomes in their 5’-UTR in *pat1* and *lsm1* mutant under osmotic stress. Among those identified mRNAs, we found the kinase Fus3. The HOG and mating pathways share several components, yet exhibit remarkable signal fidelity; hyperosmotic stress does not promote mating, and mating pheromones do not activate Hog1 [76]. To coordinate the two signals, the osmotic stress pathway limits pheromone signaling in different ways: delaying the expression of pheromone-induced genes, and promoting phosphorylation of Rck2 and Ste50 [77], to postpone its responsiveness. This suggests that the control of the translation of mRNAs related with pheromone response through Pat1 and Lsm1 might be another way of limiting cross-talk with this pathway. Altogether, our study shows that the Lsm1-7/Pat1 complex thus has a role in the translational induction regulation of stress proteins, and suggests that it regulates the accumulation of specific groups of proteins to coordinate and moderate the osmotic stress response.

### The translation repression mediated by Pat1 is dependent of Lsm1 and probably of the entire Lsm1-7/Pat1 complex

In this study, we corroborate a role for Pat1 and Lsm1 as translation repressors. As previously shown under other stress conditions such as glucose deprivation and amino acid starvation [47,78], *pat1* mutants also fail to inhibit global translation under moderate osmotic stress (S5 Fig). Moreover *pat1* and *lsm1* mutants both show a high proportion of polysomes (P/FM) when the global translation is recovered after 30 min of osmotic stress (Fig 3A). Additionally, we observe an abnormally high association of specific mRNAs to polysomes in *pat1* and *lsm1* mutants under osmotic stress (Fig 3B). Importantly, those ribosomes are able to run off mRNAs in assays done without CHX, indicating that they are not stalled and probably are in active translation (Fig 3A and 3B, second column). In addition, the *pat1* and *lsm1* mutants exhibit almost identical behaviors in our experiments where ribosome association is tested such as polysome profiles and 5P-Seq (Figs 3 and 5). Based on all these results, we propose that the translation repression mediated by Pat1 is dependent of the entire Lsm1-7/Pat1 complex, in contrast to a model where Lsm1-7 is recruited to the mRNA after Pat1 inhibits translation initiation [72]. This is supported by the fact that the Pat1 mid-domain and C-terminal domain are essential for translation repression [72] and it is through the C-terminal domain that Pat1 interacts with Lsm2-3 [44,71]. In addition, the integrity of the Lsm1-7/Pat1 complex is required for the recognition of oligoadenylated tails and total RNA binding activity, and it is in the context of a complex that Pat1 directly interacts with RNA [79]. An alternative but not mutually exclusive view is that the phenotypes in *lsm1Δ* mutants are due to depletion of Pat1 from the cytoplasm, as Pat1 is known to localize in the nucleus in such mutants [80].

Our 5P-Seq analysis of the 5’-phosphorylated mRNA co-translational degradation intermediates, where only the properly decapped mRNAs are analyzed, shows ribosome overprotection in the 5’-UTRs in *pat1* and *lsm1* mutants (Fig 5). That only this subset of mRNAs is analyzed is an inherent limitation of this technique [52]. As that protection is upstream of the translation start, this cannot reflect ongoing translation, but could mean that these mRNAs are more charged with preinitiation complexes in the absence of Lsm1 or Pat1. Sequencing of 5’ degradation intermediates has been shown to be capable of identifying binding of protein complexes to the 5’-UTR of mRNAs [81,82]. Considering previous observations reporting about Pat1, such as its association with ribosomes through its N-terminal domain, its sedimentation rate consistent with interactions with the 48S complexes, and its reduction of translation initiation by limiting the formation of a 48S preinitiation complex [72,83] rather than through a global decapping defect, the high rate of translation of *pat1* and *lsm1* mutants in our work may be interpreted as a consequence of maintaining a high level of 40S ribosomal subunit recruitment to mRNAs. However, increased binding of other proteins or complexes than 48S complexes cannot be excluded. It should be noted that in mutants defective in the Lsm1-7/Pat1 complex, poly(A) tails are shorter [42]. This could mean decreased Pab1 binding in the 3’-UTR, which could indirectly affect recruitment of other proteins to the 5’-end of an actively translating mRNA. Numerous factors determine the dynamics in translation between different mRNAs, but initiation is rate limiting for most of them [84–86]. Initiation rates are dependent on the accessibility of ribosomes to the start codon and their loading onto mRNA [87], and this is in competition with the mRNA degradation machinery to be loaded.

We thus propose that the Lsm1-7/Pat1 complex associates with stress response mRNAs, modulating their translation through limiting ribosomal access, which adds another level to the adaptability of gene regulation during rapidly changing conditions, to promote cell survival and fitness.

## Materials and methods

### Growth conditions

Unless indicated, cells were grown at 30°C till mid-log phase (OD_600_ 0.4–0.5) in synthetic medium supplemented with appropriate amino acids and 2% glucose as the carbon source. To induce osmotic stress, KCl was added to the culture as specified in the Figure legends.

### Construction of yeast strains by molecular genetics

The genotypes of all yeast strains used in this study are listed in S7 Table. The *pat1-hphNT* deletion strains were generated as described [88].

The specific MS2 loop-tagged mRNAs were generated as described previously [31]. For each gene to be tagged, a cassette *loxP*::*Sphis5*^*+*^::*loxP*::*MS2L* was obtained by PCR using pLOSHIS5MS2L as a template (kind gift from Jeffrey E. Gerst, Weizmann Institute of Science, Rehovot, Israel) [31] and the oligonucleotides listed in S8 Table. PCR products were transformed into yeast and positive transformants were selected growing on SC plates lacking histidine. To confirm integration, genomic DNA extracted from single colonies were analyzed by both PCR and sequencing (using oligos complementary to the coding region before the MS2 loops and 3’-UTR after the MS2 loops, see S8 Table). To remove the *Sphis5*^*+*^ marker, positive colonies were transformed with pSH47 (*URA3* marker), and Cre recombinase expression was induced by growing transformed cells in YP rich medium containing 2% galactose for 2 h. Yeast clones carrying *loxP*::*MS2L* integration were selected by the loss of ability to grow on SC plates lacking histidine, and verified by PCR. Finally, the loss of pSH47 was promoted by growing the final positive transformants in YPD medium for 3 days and selecting for the ability to grow in SC plates containing 100 mg/l 5-fluorouracil.

### Fluorescence microscopy

To visualize the intracellular localization of the mRNA-MS2L, expression of GFP fusion protein was induced by methionine depletion (1 h) under non-stressed or osmotic stress conditions (30 min, 0.6 M KCl). Where indicated, CHX was added to 100 μg/ml final concentration, 20 min prior to sampling in non-stressed conditions or prior to addition of KCl. After the incubation time, cells were fixed with 4% formaldehyde and stored at 4°C until analysis. The images were captured in a Zeiss Axio Observer fluorescence microscope and processed with ImageJ software (NIH). Cells with granules were counted using at least 200 cells for each mRNA. The number of granules per cell was counted using at least 60 cells for each mRNA in duplicate. Additionally, for the co-localization of the mRNA-MS2L with the PBs, cells were transformed with Dcp2-RFP plasmid [37].

### *In vivo* capture of RNPs associated with specific mRNA species

For the *in vivo* capture of specific RNPs [39], strains expressing a specific mRNA-MS2L (the background strain not expressing an mRNA-MS2L was included as a negative control) were transformed with the plasmid pMS2CP-GFP [31], which expresses the MS2 coat protein (MS2-CP) fused with GFP under the *MET25* promoter (provided by Jeffrey E Gerst). Yeast cells were grown in 600 ml of SC liquid medium lacking histidine with constant shaking at 30°C until OD_600_ 1.0 was reached. Cells were collected by centrifugation, washed once with SC medium lacking methionine and, transferred to SC medium lacking methionine during 1 h at 30°C. To induce osmotic stress, KCl was added to 0.6 M KCl after 30 min of transferring the cells and incubated for another 30 min.

Cells were collected by centrifugation and washed once with PBS buffer lacking Ca^2+^ and Mg^2+^. Crosslinking was done by resuspending cells in PBS buffer containing 0.05% formaldehyde and incubating at room temperature for 15 min with slow agitation. The cross-linking reaction was terminated by adding glycine solution (pH 8.0) to 0.125 M and incubating for 5 min with slow shaking. Fixed cells were pelleted, washed with ice-cold PBS, quick-frozen in dry ice PBS and kept at -80°C until used. Cell pellets were resuspended in 1 ml per 100 OD_600_ units of ice cold lysis buffer [20 mM Tris-HCl pH 7.5, 150 mM NaCl, 1.8 mM MgCl_2_, 1 mM DTT, 80 U/ml RNasin (Promega), 1 × protease inhibitor (Complete, Mini, EDTA-free, Roche), 0.2% Triton X-100] and aliquoted in 0.5 ml/tube. An equal volume of 0.5 ml of glass beads was added to each tube and cell were disrupted in a FastPrep device (Bio101), at intensity setting 0.5 for 3 rounds of 30 s at 4°C. Glass beads and cell debris were sedimented at RCF 1700 for 1 min at 4°C, and the supernatants were transferred to a new microcentrifuge tube. To clear the supernatant, a second centrifugation at RCF 15300 for 15 min at 4°C was done. Afterwards, the aliquots from the same sample were collected together and the protein concentration was determined using the Bicinchoninic Acid (BCA) assay (Pierce). An aliquot was kept as reference input sample.

For RNP capture, 30 mg of total protein was incubated with 100 μl of GFP-Trap beads (ChromoTek), washed and prepared for RNA manipulation following the manufacturer’s specifications, and 100 μg/ml of yeast tRNA (Sigma, R8508), for 3 h at 4°C with gentle agitation. After the RNP capture incubation, samples were washed 5 times with 1 × lysis buffer, and finally RNP complexes were eluted from the beads by adding 120 μl of 1 × cross-link reversal buffer (50 mM Tris-HCl pH 7.0, 5 mM EDTA, 10 mM DTT, 1% SDS) and incubating for 1 h at 70°C with agitation. 20 μl of eluted sample was kept for RNA determination and the remaining volume was used for western blot or LC-MS/MS analysis. Three independent replicate experiments were performed, for each tagged and untagged strain.

### *In vivo* capture of RNPs associated with specific GFP tagged proteins

Pull-downs of RNPs using specific GFP tagged proteins [89] were done following a similar protocol described above but with the following modifications. For the RNP capture, 300 μg of total protein were incubated with 20 μl 50% GFP-Trap beads slurry. Elution from the beads was done in 100 μl of 1 × cross-link reversal buffer. 80 μl of eluted samples was used for RNA determination by qPCR. Three independent experiments were done for each strain.

### Proteomics and data analysis

Protein samples were dried in a vacuum centrifuge after determination of protein concentration by the Qubit protein assay (ThermoFisher Scientific). After resuspension in SDS/PAGE sample buffer, 9 μg of protein from each sample were fractionated in a 12% polyacrylamide/SDS gel, and the gel was stained with colloidal Coomassie Blue. Afterwards, the bait protein band was excised separately and the remainder of each gel lane divided into three pieces. Protein digestion was performed as described elsewhere [90], and the resulting peptide mixture was dried in a vacuum centrifuge and resuspended in 0.1% trifluoroacetic acid (TFA), 2% acetonitrile (ACN); 20 μl for the bait protein band and 9 μl for the other gel pieces.

Mass spectrometry (LC-MS/MS) analysis was performed by loading 5 μl of tryptic peptides from each digestion mixture onto a trap column (NanoLC Column, 3 μm C18-CL, 75 μm × 15 cm; Eksigen), desalted with 0.1% TFA for 10 min at a flow rate of 2 μl/min, and transferred onto an analytical column (LC Column, 3 μm C18-CL, 75 μm × 12 cm, Nikkyo) equilibrated in 0.1% formic acid (FA) in 5% ACN. Elution was carried out with a linear gradient of 5% to 40% ACN (0.1% FA in ACN) at a flow rate of 300 nl/min. The gradient length was 45 min for the peptides generated from the bait protein band and 120 min for the peptides derived from the other gel pieces. Mass spectrometry analysis was performed with a nanoESI qQTOF (5600 TripleTOF, AB Sciex) mass spectrometer. The instrument was operated in data-dependent acquisition mode, in which a 250 ms TOF MS scan from 350–1250 m/z, was followed by 50 ms product ion scans from 100–1500 m/z on the 50 most intense 2–5 charged ions.

MS/MS data were processed with ProteinPilot v4.5 (AB Sciex). Peak lists were generated from the instrument wiff files using ProteinPilot default parameters. The peak lists derived from the three gel pieces of each sample were combined to perform a single search whereas the peak list obtained from the bait protein band was used on a separate search. Protein identification was performed with the Paragon algorithm within ProteinPilot software using the UniProt database. The following parameters were used: trypsin specificity, cys-alkylation, no taxonomy restriction, and the search effort set to thorough.

### Protein identification and quantification

The number of peptides assigned to each protein with 95% confidence or higher as well as the chromatographic intensity of each peptide are part of the ProteinPilot output. Both parameters were used to estimate protein abundance. The first quantitative method was based on the top three most intense peptides [91]. For the second quantitative method, the number of peptides assigned to each protein normalized by its molecular weight was used, which provides similar results to the emPAI calculation [92]. Two search results for each sample were obtained, one for the bait protein band and another for the remainder of the SDS/PAGE gel lane. Thus, four sets of quantitative values for each sample were generated, log transformed and normalized by the median of each set. The two quantitative sets based on peptide count were combined, but those based on peak intensity were treated individually. Three biological replicates were analyzed for each condition, and only proteins with quantitative values in at least two replicates were considered. Statistical significance of the difference relative to the untagged strain was calculated with a Student’s t-test analysis. Since proteins considered candidates to be differentially abundant were to be validated by a different set of experiments, no FDR correction was used. Differentially abundant proteins were selected as follows: only the proteins with minimum 2 values in at least one mRNA-MS2L strain and significant p-value (< 0.05) were included in the following sorting. For the subsequent filtering, the average of the normalized values from the 3 biological replicates, ratios of mRNA-MS2L/untagged strain and the ratio mRNA-MS2L (strain A)/mRNA-MS2L (strain B) were calculated.

### Analysis of steady-state mRNA levels and mRNA stability

Total RNA extraction from cells was carried out using phenol-chloroform extraction and ethanol precipitation as previously described [15]. Samples from pull-downs were treated for 30 min at 37°C with 0.2 μg/μl Proteinase K. RNA was extracted once with phenol:chloroform:isoamyl alcohol (25:24:1) and once with chloroform:isoamyl alcohol (24:1), and precipitated with 0.3 M sodium acetate, 2 volumes of 96% ethanol and 0.08 μg/μl glycogen. To extract the RNA from the polysomal fractions, 16 mM EDTA and 0.4% SDS were added, one extraction with phenol:chloroform:isoamyl alcohol (25:24:1) was performed, and RNA was precipitated twice, once with cold 96% ethanol and next with 2.5 M LiCl.

Afterwards, RNA was treated with DNase I (Thermo Fisher) according to the manufacturer’s protocol. cDNA was synthesized in 20 μl reactions containing the DNase I treated RNA, 5 μM of oligo(dT) (Thermo Fisher), 200 units/μl of M-MLV RT (Thermo Fisher), 1 × First Strand Buffer, 10 mM DTT, and 0.8 mM dNTPs. qPCR was performed in a reaction final volume of 10 μl using Eva Green (Solis) for fluorescent labeling, 2 μl cDNA, and 0.2 μM of the corresponding oligonucleotides (S8 Table). Real-time PCR reactions were performed under the following conditions: 95°C for 15 min to activate the polymerase, followed by 40 cycles of 10 s at 95°C, 20 s at 60°C, and 10 s at 72°C. At the end of the amplification cycles, a melting curve analysis was conducted to verify the specificity of the reaction. For each analyzed primer pair, a negative control was included and a standard curve was made with serial dilutions of cDNA samples pool (1/2, 1/5, 1/10, 1/25, 1/50, 1/100 and 1/500).

For measurement of mRNA stability, transcription was stopped by addition of 1,10-phenantroline to 100 μg/ml, and samples were taken at different time points thereafter (0, 5, 10, 15, 25, 35 and 45 min) [35]. RNA was extracted and analyzed as described above.

### Polysome analysis

Polyribosome analysis was performed as previously described [20]. Cells grown to mid-log phase were treated with 0.1 mg/ml CHX and collected by centrifugation. Cells pellets were lysate in lysis buffer (20 mM Tris-HCl, pH 8.0, 140 mM KCl, 5 mM MgCl_2_, 0.5 mM DTT, 1% Triton X-100, 0.1 mg/ml CHX, and 0.5 mg/ml heparin) by bead bashing. Finally, glycerol was added to the supernatant to a final concentration of 5%, and extracts were stored at -80°C. Samples of 10–12 A_260_ units were loaded onto 10 – 50% sucrose gradients and were separated by ultracentrifugation for 2 h and 40 min at 35,000 rpm in a Beckman SW41Ti rotor at 4°C. Gradients were then fractionated and recorded using Density Gradient Fractionation System and Isco UA-6 ultraviolet detector (Teledyne Isco, Lincoln, NE, USA). For runoff experiments, cells and samples were handled as described above but CHX was omitted in both the lysis buffer and the gradient solutions.

### Detection of specific proteins by Western blot analysis and fluorescence emission

Western blot was performed using standard protocols. Antibodies used were mouse anti-GFP (1:1000, Roche), rat α-She2 (1:50, kind gift from Ralf Jansen, University of Tübingen, Germany), α-mouse (1:5000, Sigma) and α-rat (1:500, Amersham) secondary antibodies conjugated to horseradish peroxidase.

Accumulation of GFP-tagged proteins was detected by recording green fluorescence emission (520 nm) in an Omega Polarstar fluorescence plate reader (LabVision). Plates were incubated at 30°C and measurements were taken automatically every 4 min for a period of 140 min. To normalize the data, OD_600_ measured from the same well at the same time than green fluorescence and, was used to remove the effect of growth differences between strains.

### Analysis of co-translational mRNA decay by global 5’P sequencing

Libraries for 5’-phosphate sequencing (5P-Seq) were prepared as specified [52] using 6 μg of DNA-free total RNA. To select 5’-P mRNA degradation intermediates, samples were directly subjected to selective ligation of a synthetic DNA/RNA oligo containing unique molecular identifiers (UMIs); samples were incubated overnight at 16°C with 20 units of T4 RNA ligase 1 (New England Biolabs) in the presence of 10 mM DNA/RNA rP5_RND oligo [52]. RNA integrity was checked by electrophoresis in 1 × TAE agarose gel and ribosomal RNA was depleted using Ribo-Zero Magnetic Gold Kit (Epicentre). PolyA-enriched RNA was fragmented at 80°C for 5 min in the presence of RNA fragmentation buffer (40 mM Tris-acetate, pH 8.1, 100 mM KOAc, 30 mM MgOAc), and reverse transcribed with Superscript II (Life Technologies) primed with random hexamers. The retrotranscription reaction was incubated for 10 min at 25°C, 50 min at 42°C and heat inactivated for 15 min at 72°C. Second strand cDNA synthesis was performed by a single PCR cycle (1 min at 98°C; 2 min at 50°C and 15 min at 72°C) using Phusion High-Fidelity PCR Master Mix with HF Buffer (New England Biolabs) and priming with BioNotI-P5-PET [52]. From this point onwards, libraries were generated as described previously [93]. Briefly, double-stranded cDNA was first purified using HighPrep beads (Magbio) and then bound to Dynabeads M-280 Streptavidin beads (Life Technologies) according to the manufacturer’s instructions. Bound DNA molecules were successively washed and subjected to repair of ends, dA addition and adaptor ligation using Nebnext DNA Library Prep Master Mix (NEB). 0.5 μl of common adapter (2.5 μM) was ligated to each sample. The common adapter was prepared by annealing P7MPX_linker_for and P7MPX_linker_rev [52]. Beads were washed and subjected to PCR amplification [30 s 98°C; 19 cycles of (20 s 98°C, 30 s 65°C, 30 s 72°C); 5 min 72°C] using Phusion High-Fidelity PCR Master Mix with HF Buffer (NEB) and 0.1 μM final concentration of PCR-PE 1.0 and the appropriate PE2_MTX [52]. Libraries were quantified by Qubit using the dsDNA HS assay kit and the size was checked using a RNA Bioanalyzer. A 5P-Seq pool was made mixing 8 ng of each amplified 5P-Seq library, and was size selected using 0.6 × - 0.9 × (v/v) HighPrep beads (Magbio) to 300 – 500 bp. The 5P-Seq pool was sequenced using an Illumina NextSeq 500 High Output Kit v2.

### Sequence analysis

We trimmed the first 8 nt off each read (containing the unique molecular identifier, UMI) and aligned the rest to the *S*. *cerevisiae* genome (R64-1-1). For mapping, we used Hisat2 with default parameters (except maximum intron size set to 2 kb). Reads with identical 5’mapping site and UMI were considered PCR duplicates, and collapsed. To compute gene-specific pausing we generated count tables for each gene for the 45 nt regions upstream. Only genes with at least 20 5P-Seq read in the defined regions were considered for the analysis. Raw and processed sequencing data are deposited at GEO with accession number GSE107250.

## Supporting information

S1 Fig Verification of genomic MS2L tagging and checking the expression of mRNA-MS2L. A) Checking the genomic tagging by PCR using primers flanking MS2 loops integration site. The presence of integrated MS2 loops was indicated by PCR amplification of a 1 kb fragment. B) Checking the expression of mRNA-MS2L by qPCR, in control conditions (without stress) and after 30 min of 0.6 M KCl. C) Time course expression of *STL1-MS2L* and *GPD1-MS2L* mRNA levels under osmotic stress expressed in log_2_ E^-ΔΔCt^. In B) and C) the level of *ACT1* housekeeping mRNA was used as a reference. D) Percentage native (wt strain) and MS2L tagged *STL1* and *GPD1* mRNAs associated to polysomes after 30 min of osmotic stress (FM, free and monosome fractions; P, polysome fraction). Average and standard error (SE) from three biological replicates are shown.
(PDF)

S2 Fig Intracellular localization of the MS2L-mRNAs. **The localization was monitored by visualization of green fluorescence emitted by MS2-CP-GFP fusion protein bound to them.** A) Fluorescence microscopy images to show localization of MS2L-mRNA after induction of the MS2-CP-GFP protein expression by methionine depletion under normal (SC met-, 1 h) and osmotic stress conditions (with and without addition of CHX). B) Quantification of the percentage of cells with granules and number of granules per cell. Where indicated, CHX was added 20 min prior to sampling (control conditions) or prior to stress (0.6 M KCl). C) Fluorescence microscopy merge images of cells expressing MS2L-mRNAs (green) and the PB marker Dcp2p (Dcp2p-RFP plasmid, red), under osmotic stress conditions (0.6 M KCl, 30 min). The percentage of granules with colocalization of green and red fluorescence is indicated beside the images. At least 100 cells were analyzed for each strain. D) Percentage of cells containing Dcp2-RFP foci in cells expressing MS2L-mRNAs, under osmotic stress conditions. Samples were taken at different time points (0, 15, and 30 min) after addition of 0.6 M KCl, and at least 100 cells were analyzed in each. The experiment was done in duplicate or triplicate, with the exception of the *ASH1-MS2L* strain. Average and standard error (SE) from the replicates are shown. Statistical analyses were performed using Student’s t-test of comparisons between strain without MS2L and MS2L-mRNAs strains, and no significant difference was found between samples (all p-values > 0.1).
(PDF)

S3 Fig Testing the RNP capture using *ASH1-MS2L* strain. A) Fusion protein (GFP) and She2 protein binding specifically to *ASH1* mRNA were detected by western blot. B) PCR detection using *ASH1* ORF primers of *ASH1-MS2L* mRNA and native *ASH1* mRNA in the tagged and untagged (BY) strains, respectively, both expressing the MS2CP-GFP fusion protein. Detection was made in extracts before the pull-down (Input, I) and in the pull-down samples (PD).
(PDF)

S4 Fig *HYP2* mRNA association to polysomes at 30 min of osmotic stress (0.6 M KCl) for wt (blue line), *pat1* (red line) and *lsm1* (green line) strains. Ratios between polysomal and sub-polysomal fraction (P/FM) is represented in the right column chart. Average and standard error (SE) from three biological replicates are shown.
(PDF)

S5 Fig **A) Polysome profiles of a time course experiment under osmotic stress (0.6 M KCl) in *pat1* mutant and wt strains.** Ratio P/FM between polysomal (P, fractions 3–8) and sub-polysomal fraction (FM, fractions 1–2) is indicated below each profile. B) Detection of specific mRNA association to the fractions along the gradient by qPCR. Ratios between polysomal and sub-polysomal fraction (P/FM) at each time for wt (blue bars) and *pat1* (red bars) strains are represented in the right column charts. For *STL1* and *GPD1* mRNAs, time 0 was not analyzable because of their low levels under non-stress conditions.
(PDF)

S6 Fig mRNA levels from GFP-tagged strains for the same genes for which the levels of tagged proteins were assayed under osmotic stress (0.6 M KCl; Fig 4), expressed as increment relative to time 0, using *ACT1* as reference mRNA. (PDF)

S7 Fig **A) Histograms showing the total number of 5P-seq reads in each reading frame in the 5’UTR (in blue, window of 80 nt before the start codon) and in coding regions (in pink, window of 80 nt from the start codon), under control conditions (without stress) and after 30 min of osmotic stress.** Number of readings are included for each bar. B) Scatter plots displaying 5P-seq values for *lsm1* mutant (Y-axis) and wt (X-axis) strains. The scatter plots represent the log_2_ ratios between window areas around the start codon (uSvsdS) or the stop codon (uEvsdE) without (upper panels) and with osmotic stress (bottom panels).
(PDF)

S1 Table Interaction of other proteins related to mRNA decay with *STL1* and *GPD1* mRNAs, data from MS experiments. (DOCX)

S2 Table Translation efficiency for single genes in wt strain and *pat1* mutant, calculated as the ratio between the areas under the curve of mRNA and protein after osmotic stress (0.6 M KCl). The area was calculated till 60 min under osmotic stress and till the levels reached a plateau, for mRNA and for protein respectively (Fig 4 and S6 Fig).
(DOC)

S3 Table Relative 5P-seq protection in the 5’UTR region in *pat1* mutants for the mRNAs for which protein synthesis is analyzed in Fig 4. (DOC)

S4 Table Mann-Whitney test of increased difference of mRNA 5P-seq coverage in mutants vs. wt in OSR vs. main group shown in Fig 5C. (DOC)

S5 Table GO term analysis (http://babelomics.bioinfo.cipf.es/) for ranked mRNAs based on their fold change of 5’-UTR ribosome accumulation in *lsm1* and *pat1* mutants relative to wt (calculated as log_2_ [uSvsdS_mutants_/uSvsdS_wt_]) in control conditions and under osmotic stress (0.6 M KCl, 30 min). (XLSX)

S6 Table Osmotic stress induced genes. Lists were generated using a compendium of several previously published mRNA data [14,34,94,95].
(XLSX)

S7 Table Yeast strains used in this study. (DOC)

S8 Table Oligonucleotides used in this study (plasmid sequence underlined). (DOC)

S1 Dataset Data for Figs 2–4. (XLSX)

S2 Dataset Data for Fig 5A. (XLSX)

S3 Dataset Data for Fig 5B and 5C and for S7B Fig. (XLSX)

S4 Dataset Data for all other supplementary figs. (XLSX)
