# Supplementary material for: The Lsm1-7/Pat1 complex binds to stress-activated mRNAs and modulates the response to hyperosmotic shock
Source: PLoS Genet. 2018 Jul 30;14(7):e1007563. doi: 10.1371/journal.pgen.1007563 (PMC6085073; doi:10.1371/journal.pgen.1007563)

Supplementary Fig. S1

Verification of genomic MS2L tagging and checking the expression of mRNA-MS2L

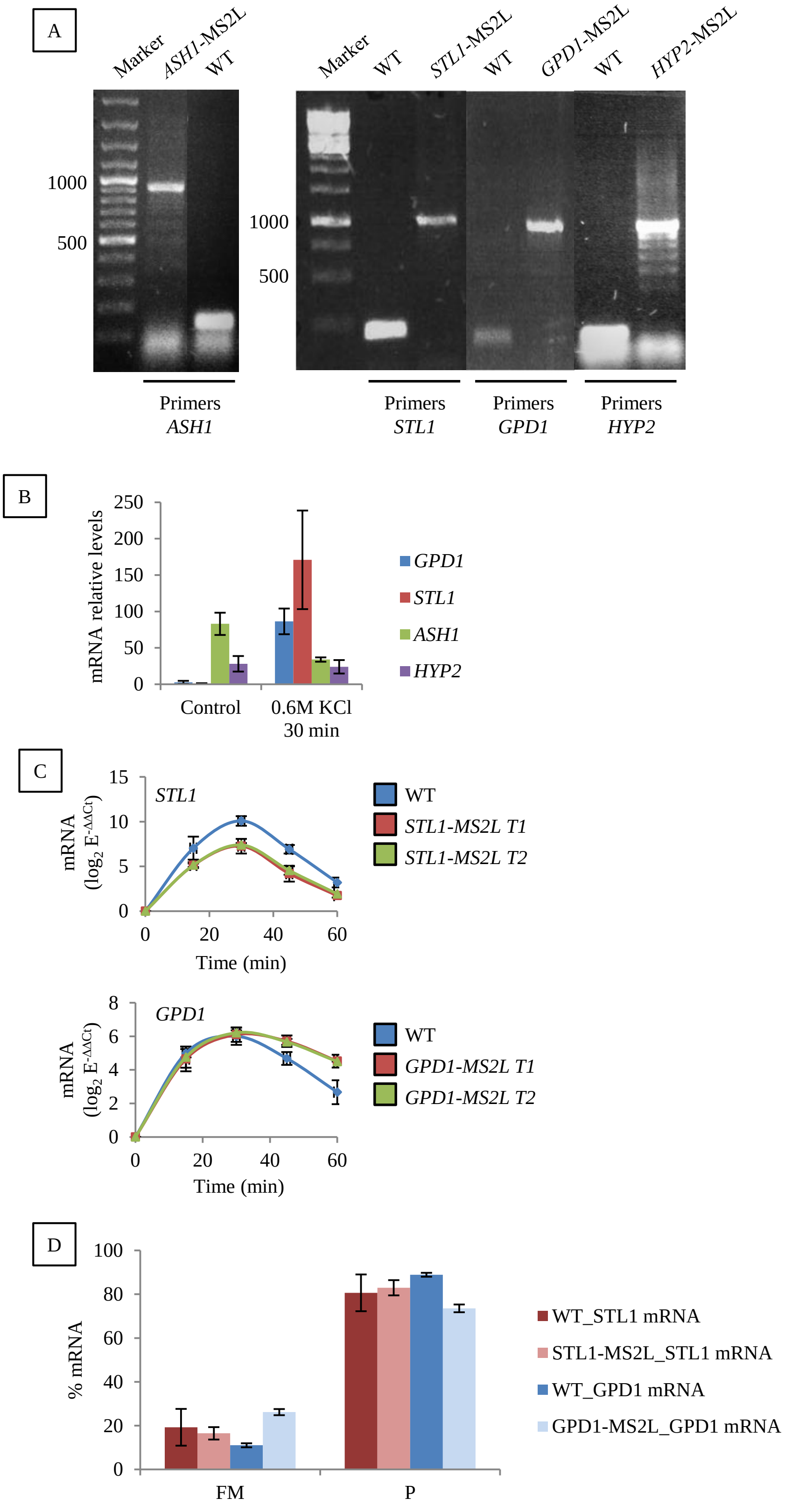

Supplement: S1 Fig — A) Checking the genomic tagging by PCR using primers flanking MS2 loops integration site. The presence of integrated MS2 loops was indicated by PCR amplification of a 1 kb fragment. B) Checking the expression of mRNA-MS2L by qPCR, in control conditions (without stress) and after 30 min of 0.6 M KCl. C) Time course expression of STL1-MS2L and GPD1-MS2L mRNA levels under osmotic stress expressed in log2 E-ΔΔCt. In B) and C) the level of ACT1 housekeeping mRNA was used as a reference. D) Percentage native (wt strain) and MS2L tagged STL1 and GPD1 mRNAs associated to polysomes after 30 min of osmotic stress (FM, free and monosome fractions; P, polysome fraction). Average and standard error (SE) from three biological replicates are shown. (PDF) [file pgen.1007563.s001.pdf]
