# Supplementary figures and images for: The Lsm1-7/Pat1 complex binds to stress-activated mRNAs and modulates the response to hyperosmotic shock
Source: PLoS Genet. 2018 Jul 30;14(7):e1007563. doi: 10.1371/journal.pgen.1007563 (PMC6085073; doi:10.1371/journal.pgen.1007563)

## Supplementary Fig. S2

### Intracellular localization of the MS2L-mRNAs

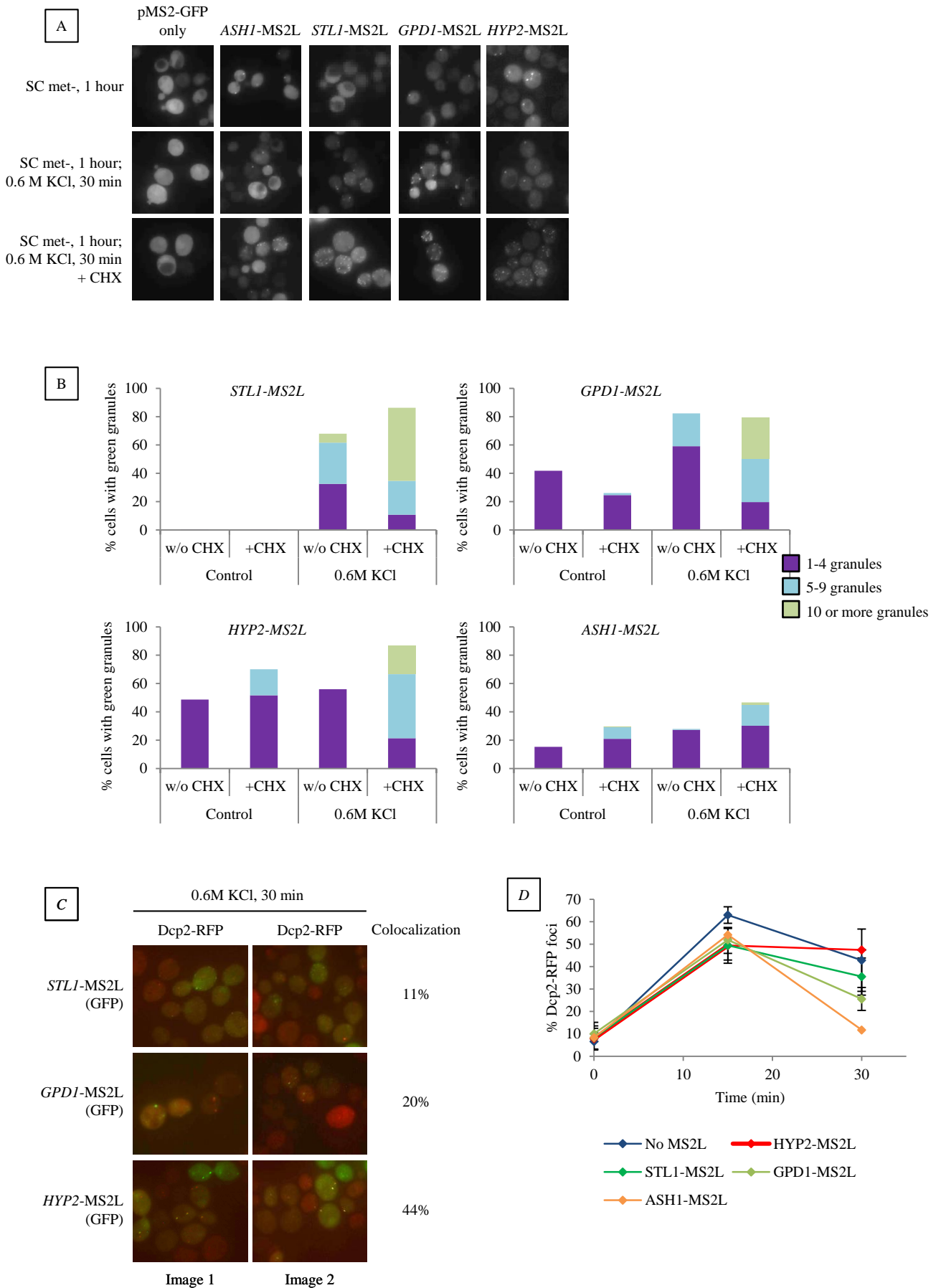

Supplement: S2 Fig — The localization was monitored by visualization of green fluorescence emitted by MS2-CP-GFP fusion protein bound to them. A) Fluorescence microscopy images to show localization of MS2L-mRNA after induction of the MS2-CP-GFP protein expression by methionine depletion under normal (SC met-, 1 h) and osmotic stress conditions (with and without addition of CHX). B) Quantification of the percentage of cells with granules and number of granules per cell. Where indicated, CHX was added 20 min prior to sampling (control conditions) or prior to stress (0.6 M KCl). C) Fluorescence microscopy merge images of cells expressing MS2L-mRNAs (green) and the PB marker Dcp2p (Dcp2p-RFP plasmid, red), under osmotic stress conditions (0.6 M KCl, 30 min). The percentage of granules with colocalization of green and red fluorescence is indicated beside the images. At least 100 cells were analyzed for each strain. D) Percentage of cells containing Dcp2-RFP foci in cells expressing MS2L-mRNAs, under osmotic stress conditions. Samples were taken at different time points (0, 15, and 30 min) after addition of 0.6 M KCl, and at least 100 cells were analyzed in each. The experiment was done in duplicate or triplicate, with the exception of the ASH1-MS2L strain. Average and standard error (SE) from the replicates are shown. Statistical analyses were performed using Student’s t-test of comparisons between strain without MS2L and MS2L-mRNAs strains, and no significant difference was found between samples (all p-values > 0.1). (PDF) [file pgen.1007563.s002.pdf]

Supplementary Fig. S3

Testing the RNP capture  
using *ASH1-MS2L* strain

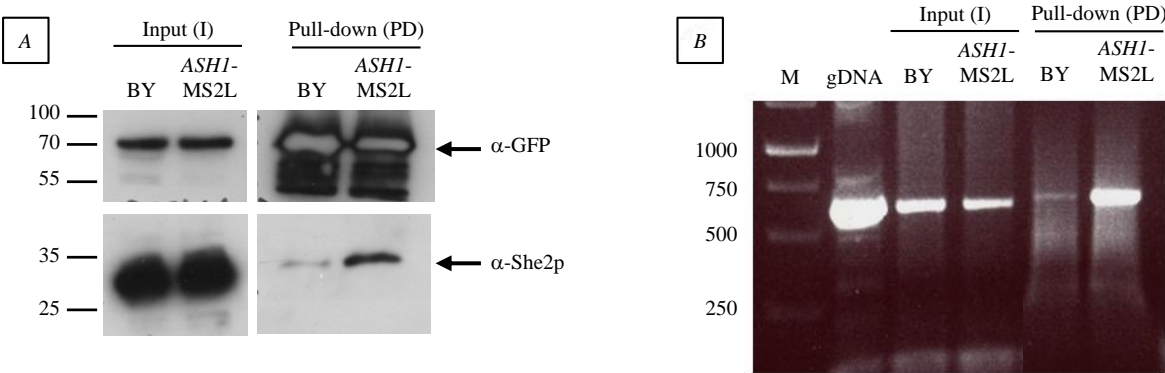

Supplement: S3 Fig — A) Fusion protein (GFP) and She2 protein binding specifically to ASH1 mRNA were detected by western blot. B) PCR detection using ASH1 ORF primers of ASH1-MS2L mRNA and native ASH1 mRNA in the tagged and untagged (BY) strains, respectively, both expressing the MS2CP-GFP fusion protein. Detection was made in extracts before the pull-down (Input, I) and in the pull-down samples (PD). (PDF) [file pgen.1007563.s003.pdf]
