# Supplementary material for: The Lsm1-7/Pat1 complex binds to stress-activated mRNAs and modulates the response to hyperosmotic shock
Source: PLoS Genet. 2018 Jul 30;14(7):e1007563. doi: 10.1371/journal.pgen.1007563 (PMC6085073; doi:10.1371/journal.pgen.1007563)

Supplementary Fig. S4

Association of *HYP2* mRNA to polysomal profiles after 30 min of 0.6 M KCl in the presence of CHX

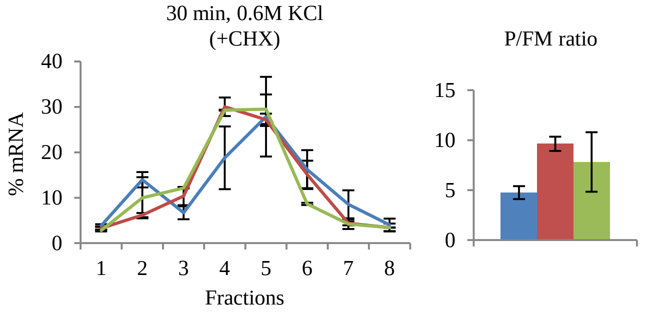

Supplement: S4 Fig — Ratios between polysomal and sub-polysomal fraction (P/FM) is represented in the right column chart. Average and standard error (SE) from three biological replicates are shown. (PDF) [file pgen.1007563.s004.pdf]
