# Supplementary material for: The Lsm1-7/Pat1 complex binds to stress-activated mRNAs and modulates the response to hyperosmotic shock
Source: PLoS Genet. 2018 Jul 30;14(7):e1007563. doi: 10.1371/journal.pgen.1007563 (PMC6085073; doi:10.1371/journal.pgen.1007563)

Supplementary Fig. S5

A) Polysome profiles of a time course experiment under osmotic stress (0.6 M KCl) in *pat1* mutant and wt strains

B) Detection of specific mRNA association to the fractions along the gradient by qPCR

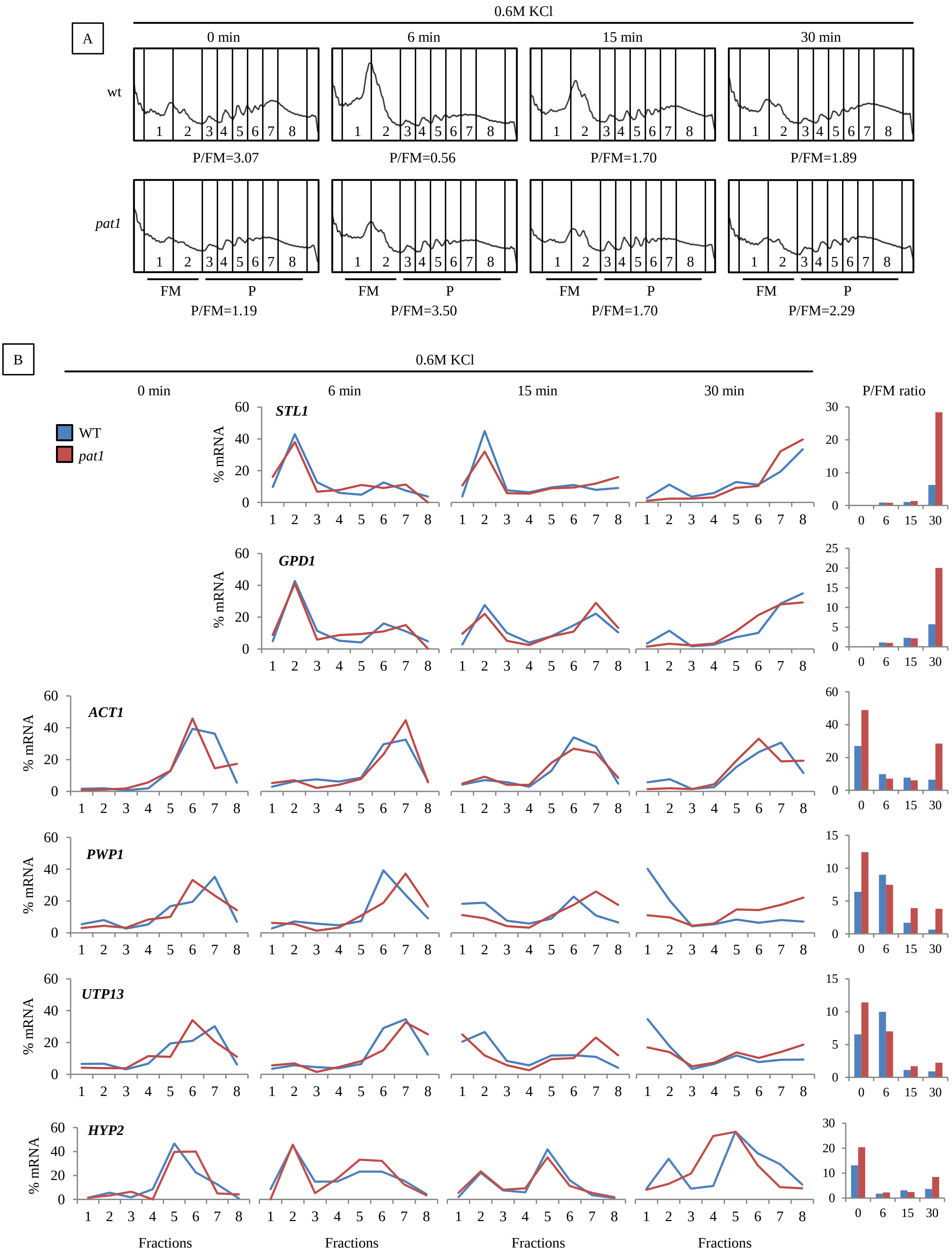

Supplement: S5 Fig — A) Polysome profiles of a time course experiment under osmotic stress (0.6 M KCl) in pat1 mutant and wt strains. Ratio P/FM between polysomal (P, fractions 3–8) and sub-polysomal fraction (FM, fractions 1–2) is indicated below each profile. B) Detection of specific mRNA association to the fractions along the gradient by qPCR. Ratios between polysomal and sub-polysomal fraction (P/FM) at each time for wt (blue bars) and pat1 (red bars) strains are represented in the right column charts. For STL1 and GPD1 mRNAs, time 0 was not analyzable because of their low levels under non-stress conditions. (PDF) [file pgen.1007563.s005.pdf]
