# Supplementary material for: The Lsm1-7/Pat1 complex binds to stress-activated mRNAs and modulates the response to hyperosmotic shock
Source: PLoS Genet. 2018 Jul 30;14(7):e1007563. doi: 10.1371/journal.pgen.1007563 (PMC6085073; doi:10.1371/journal.pgen.1007563)

Supplementary Fig. S6

mRNA levels from GFP-tagged strains for the same genes for which the levels of tagged proteins were assayed under osmotic stress in Fig. 4

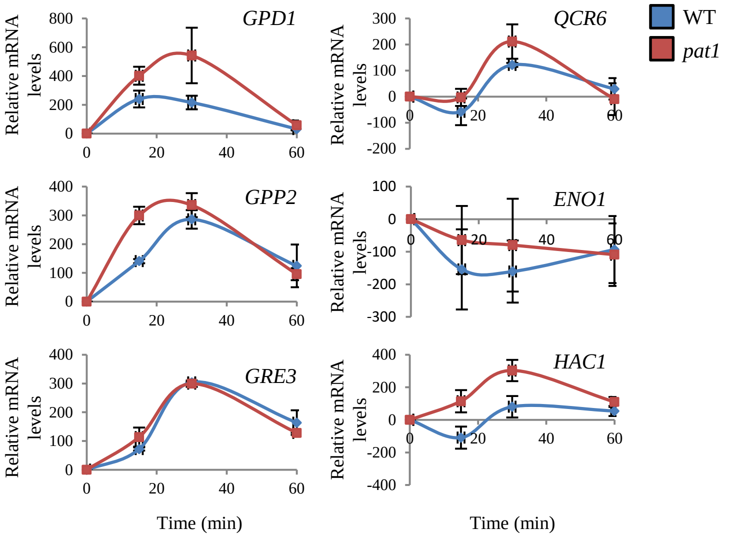

Supplement: S6 Fig — (PDF) [file pgen.1007563.s006.pdf]
