# Supplementary material for: The Lsm1-7/Pat1 complex binds to stress-activated mRNAs and modulates the response to hyperosmotic shock
Source: PLoS Genet. 2018 Jul 30;14(7):e1007563. doi: 10.1371/journal.pgen.1007563 (PMC6085073; doi:10.1371/journal.pgen.1007563)

Supplementary Fig. S7

A) Histograms showing the total number of 5P-seq reads in each reading frame in the 5'UTR
B) Scatter plots displaying 5P-seq values for *lsm1* mutant (Y-axis) and wt (X-axis) strains

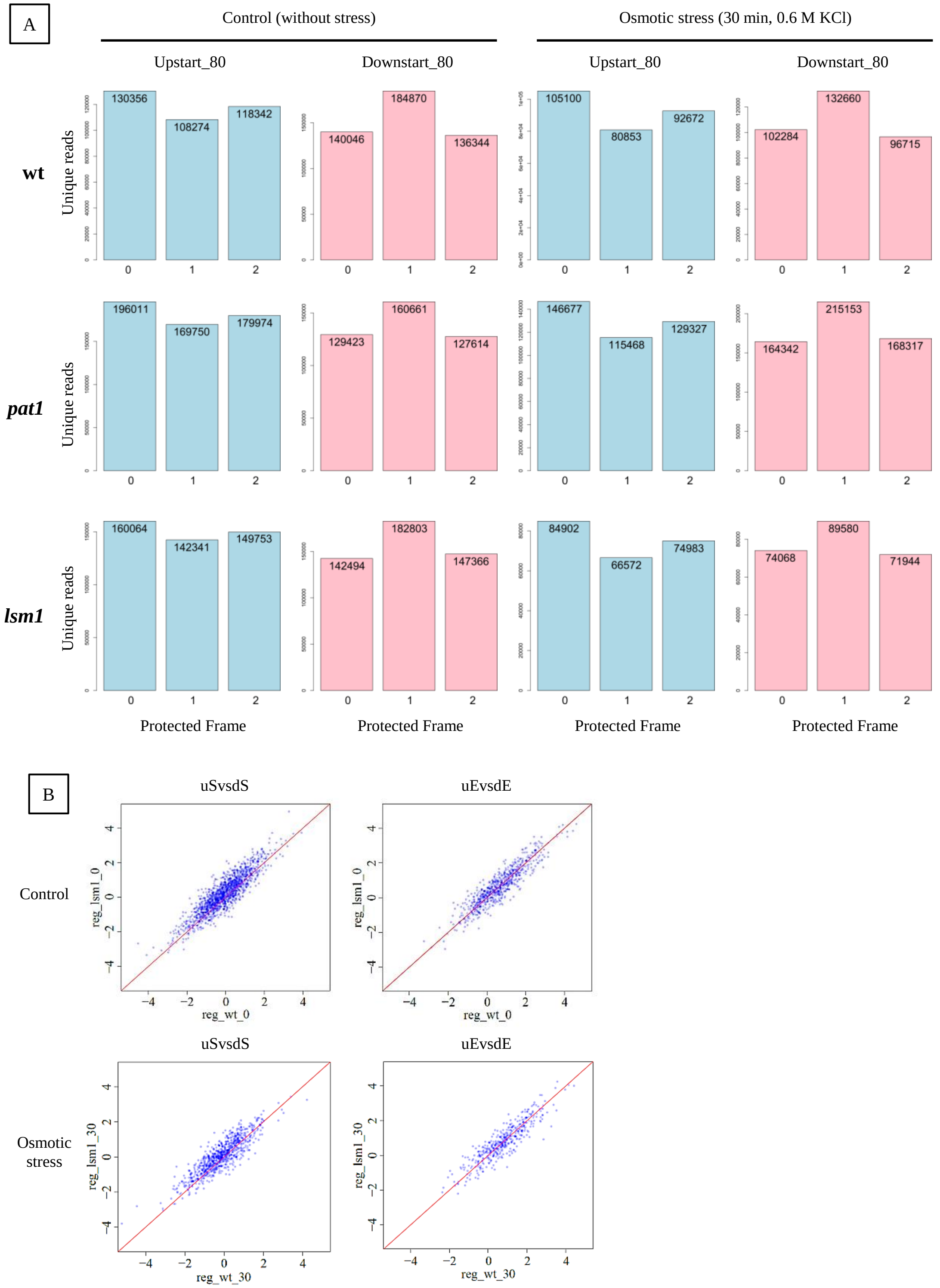

Supplement: S7 Fig — A) Histograms showing the total number of 5P-seq reads in each reading frame in the 5’UTR (in blue, window of 80 nt before the start codon) and in coding regions (in pink, window of 80 nt from the start codon), under control conditions (without stress) and after 30 min of osmotic stress. Number of readings are included for each bar. B) Scatter plots displaying 5P-seq values for lsm1 mutant (Y-axis) and wt (X-axis) strains. The scatter plots represent the log2 ratios between window areas around the start codon (uSvsdS) or the stop codon (uEvsdE) without (upper panels) and with osmotic stress (bottom panels). (PDF) [file pgen.1007563.s007.pdf]
