# Supplementary material for: The Lsm1-7/Pat1 complex binds to stress-activated mRNAs and modulates the response to hyperosmotic shock
Source: PLoS Genet. 2018 Jul 30;14(7):e1007563. doi: 10.1371/journal.pgen.1007563 (PMC6085073; doi:10.1371/journal.pgen.1007563)
Supplement: S1 Table — (DOCX) [file pgen.1007563.s008.docx]

**S1 Table.** Interaction of other proteins related to mRNA decay with *STL1* and *GPD1* mRNAs, data from MS experiments.

|  |  |  | Enrichment peptides | | | | Enrichment TopAny | | | |
| --- | --- | --- | --- | --- | --- | --- | --- | --- | --- | --- |
| Gene | Systematic name | Function | *GPD1/ASH1* | *STL1/ASH1* | *GPD1/HYP2* | *STL1/HYP2* | *GPD1/ASH1* | *STL1/ASH1* | *GPD1/HYP2* | *STL1/HYP2* |
| *LSM3* | YLR438C-A | Decay | - | - | - | - | - | - | - | - |
| *LSM7* | YNL147W | Decay | 1,56 | 0,83 | 10,24 | 9,50 | 2,26 | -1,26 | 13,26 | 9,74 |
| *LSM8* | YJR022W | Decay | - | - | - | - | - | - | - | - |
| *DCP1* | YOL149W | Decapping, decay | 1,84 | 1,49 | 1,11 | 0,77 | 1,30 | 1,54 | 0,21 | 0,46 |
| *DCP2* | YNL118C | Deccaping, decay | 1,38 | 1,38 | 1,64 | 1,64 | 0,47 | 0,34 | 1,30 | 1,18 |
| *DHH1* | YDL160C | Decay | -0,02 | -0,04 | 0,28 | 0,26 | 0,01 | -0,05 | 0,92 | 0,86 |
| *XRN1* | YGL173C | Decay | 0,74 | 1,21 | 0,11 | 0,58 | 0,67 | 1,21 | -0,01 | 0,53 |
| *CCR4* | YAL021C | Deadenylation, decay | -0,86 | 0,17 | -0,56 | 0,48 | -0,02 | 0,31 | -0,53 | -0,20 |
| *CAF1* | YNR052C | Deadenylation, decay | - | - | - | - | - | - | - | - |
| *CAF40* | YNL288W | Deadenylation, decay | 0,19 | -0,40 | 0,28 | -0,32 | 0,61 | -0,50 | 0,43 | -0,68 |
| *CAF130* | YGR134W | Deadenylation, decay | - | - | - | - | - | - | - | - |
| *CAF4* | YKR036 | Deadenylation, decay | 0,97 | 0,41 | -0,04 | -0,60 | - | - | - | - |
| *CAF16* | YFL028C | Deadenylation, decay | - | - | - | - | - | - | - | - |
| *NOT1* | YCR093W | Deadenylation, decay | -0,62 | -0,72 | -0,34 | -0,44 | -0,29 | -0,34 | -0,35 | -0,40 |
| *NOT2* | YDL165W | Deadenylation, decay | -0,26 | -0,24 | 1,20 | 1,22 | 2,47 | 2,30 | 1,17 | 1,00 |
| *NOT3* | YIL038C | Deadenylation, decay | -1,42 | 0,31 | 5,35 | 7,09 | -1,38 | -2,07 | 10,67 | 9,98 |
| *NOT4* | YER068W | Deadenylation, decay | -0,77 | 1,26 | -0,36 | -0,43 | 1,05 | 0,89 | -2,36 | -2,52 |
| *NOT5* | YPR072W | Deadenylation, decay | -0,37 | -1,31 | -7,18 | 1,06 | -0,89 | 1,44 | 10,84 | 13,17 |
| *RPB4* | YJL140W | Transcription, decay | - | - | - | - | 1,25 | -1,82 | 1,16 | -1,91 |
| *RPB7* | YDR404C | Transcription, decay | 0,97 | 0,87 | 1,28 | 1,18 | 4,92 | 4,51 | 0,92 | 0,51 |

*statistically significant^$^ log_2_ ratio >1.5 (3-fold change)
