# Supplementary material for: The Lsm1-7/Pat1 complex binds to stress-activated mRNAs and modulates the response to hyperosmotic shock
Source: PLoS Genet. 2018 Jul 30;14(7):e1007563. doi: 10.1371/journal.pgen.1007563 (PMC6085073; doi:10.1371/journal.pgen.1007563)
Supplement: S2 Table — The area was calculated till 60 min under osmotic stress and till the levels reached a plateau, for mRNA and for protein respectively (Fig 4 and S6 Fig). (DOC) [file pgen.1007563.s009.doc]

**S2 Table.** Translation efficiency for single genes in wt strain and *pat1* mutant, calculated as the ratio between the areas under the curve of mRNA and protein after osmotic stress (0.6 M KCl). The area was calculated until 60 min under osmotic stress and until the levels reached a plateau, for mRNA and for protein respectively (Fig 4 and S6 Fig).

|  | Ratio prot/RNA | | |
| --- | --- | --- | --- |
|  | *GPD1* | *GPP2* | *GRE3* |
| wt | 17.5 | 17.2 | 27.8 |
| *pat1* | 29.4 | 86.5 | 19.4 |
| Ratio *pat1*/wt | 1.7 | 5.0 | 0.7 |
