# Supplementary material for: The Lsm1-7/Pat1 complex binds to stress-activated mRNAs and modulates the response to hyperosmotic shock
Source: PLoS Genet. 2018 Jul 30;14(7):e1007563. doi: 10.1371/journal.pgen.1007563 (PMC6085073; doi:10.1371/journal.pgen.1007563)
Supplement: S3 Table — (DOC) [file pgen.1007563.s010.doc]

**S3 Table.** Relative protection in the 5’UTR region in *pat1* mutants for the mRNAs for which protein synthesis is analyzed in Fig 4.

|  | 5’UTR protection *pat1* relative to wt | | | |
| --- | --- | --- | --- | --- |
|  | Time 0 | | Time 30 min  0.6 M KCl | |
|  | uSvsdS | uSvsddS | uSvsdS | uSvsddS |
| *GPD1* | -0.16 | 0.40 | 0.50 | -0.18 |
| *GPP2* | -0.46 | 1.45 | 0.32 | 0.52 |
| *GRE3* | -0.76 | -0.73 | -0.12 | 0.36 |
| *HAC1* | -0.19 | -0.38 | 0.03 | -0.06 |
| *QCR6* | -0.21 | -0.27 | 0.03 | -0.69 |
| *ENO1* | 0.92 | 0.83 | 0.53 | 0.60 |
