# Supplementary material for: The Lsm1-7/Pat1 complex binds to stress-activated mRNAs and modulates the response to hyperosmotic shock
Source: PLoS Genet. 2018 Jul 30;14(7):e1007563. doi: 10.1371/journal.pgen.1007563 (PMC6085073; doi:10.1371/journal.pgen.1007563)
Supplement: S4 Table — (DOC) [file pgen.1007563.s011.doc]

**S4 Table**. Mann-Whitney U test applied to the increment of cumulative distribution of 5P-seq coverage between wt and mutants in OSR group against the rest of mRNAs shown in Fig. 5.

|  |  | N | | Mean Rank | |  |  |
| --- | --- | --- | --- | --- | --- | --- | --- |
|  |  | OSR | Not OSR | OSR | Not OSR | Mann-Whitney U | p-value (2-tailed) |
| uSvsdS | Control | 244 | 1508 | 938.04 | 866.54 | 168961 | 0.041* |
|  | Osmotic stress | 261 | 750 | 514.36 | 503.09 | 95693 | 0.591 |
| uEvsdE | Control | 94 | 654 | 383.17 | 373.25 | 29923 | 0.677 |
|  | Osmotic stress | 111 | 375 | 232.74 | 246.69 | 19618 | 0.358 |

(p-value < 0.05*)
