# Supplementary material for: The Lsm1-7/Pat1 complex binds to stress-activated mRNAs and modulates the response to hyperosmotic shock
Source: PLoS Genet. 2018 Jul 30;14(7):e1007563. doi: 10.1371/journal.pgen.1007563 (PMC6085073; doi:10.1371/journal.pgen.1007563)
Supplement: S7 Table — (DOC) [file pgen.1007563.s014.doc]

**S7 Table. Yeast strains used in this study.**

| Strain | Genotype | Source |
| --- | --- | --- |
| BY4741 | MATa *his3Δ1 leu2Δ0 met15Δ0 ura3Δ0* | Euroscarf |
| *pat1* | BY4741; *pat1*::kanMX4 | Euroscarf |
| *lsm1* | BY4741; *lsm1*::kanMX4 | Euroscarf |
| *GPD1-MS2L* | BY4741; *GPD1::loxP::MS2L::GPD1^3’UTR^* | This study |
| *STL1-MS2L* | BY4741; *STL1::loxP::MS2L::STL1^3’UTR^* | This study |
| *ASH1-MS2L* | BY4741; *ASH1::loxP::MS2L::ASH1^3’UTR^* | This study |
| *HYP2-MS2L* | BY4741; *HYP2::loxP::MS2L::HYP2^3’UTR^* | This study |
| Pat1p-GFP | BY4741; *PAT1::GFP::HiS3MX* | [Huh, 2003 #3707] |
| Lsm1p-GFP | BY4741; *LSM1::GFP::HiS3MX* | [Huh, 2003 #3707] |
| Lsm3p-GFP | BY4741; *LSM3::GFP::HiS3MX* | [Huh, 2003 #3707] |
| Lsm4p-GFP | BY4741; *LSM4::GFP::HiS3MX* | [Huh, 2003 #3707] |
| Lsm7p-GFP | BY4741; *LSM7::GFP::HiS3MX* | [Huh, 2003 #3707] |
| His2p-GFP | BY4741; *HIS2::GFP::HiS3MX* | [Huh, 2003 #3707] |
| Clb2p-GFP | BY4741; *CLB2::GFP::HiS3MX* | [Huh, 2003 #3707] |
| Gpd1p-GFP | BY4741; *GPD1::GFP::HiS3MX* | [Huh, 2003 #3707] |
| Gpp2p-GFP | BY4741; *GPP2::GFP::HiS3MX* | [Huh, 2003 #3707] |
| Gre3p-GFP | BY4741; *GRE3::GFP::HiS3MX* | [Huh, 2003 #3707] |
| Eno1p-GFP | BY4741; *ENO1::GFP::HiS3MX* | [Huh, 2003 #3707] |
| Qcr6p-GFP | BY4741; *QCR6::GFP::HiS3MX* | [Huh, 2003 #3707] |
| Hac1p-GFP | BY4741; *HAC1::GFP::HiS3MX* | [Huh, 2003 #3707] |
| *pat1* Gpd1p-GFP | Gpd1p-GFP; *pat1*:: *hphNT* | This study |
| *pat1* Gpp2p-GFP | Gpp2p-GFP; *pat1*:: *hphNT* | This study |
| *pat1* Gre3p-GFP | Gre3p-GFP; *pat1*:: *hphNT* | This study |
| *pat1* Eno1p-GFP | Eno1p-GFP; *pat1*:: *hphNT* | This study |
| *pat1* Qcr6p-GFP | Qcr6p-GFP; *pat1*:: *hphNT* | This study |
| *pat1* Hac1p-GFP | Hac1p-GFP; *pat1*:: *hphNT* | This study |
