# Supplementary material for: The Lsm1-7/Pat1 complex binds to stress-activated mRNAs and modulates the response to hyperosmotic shock
Source: PLoS Genet. 2018 Jul 30;14(7):e1007563. doi: 10.1371/journal.pgen.1007563 (PMC6085073; doi:10.1371/journal.pgen.1007563)
Supplement: S8 Table — (DOC) [file pgen.1007563.s015.doc]

**S8 Table. Oligonucleotides used in this study.** (plasmid sequence underlined).

| Name | Sequence | Use |
| --- | --- | --- |
| PAT1-S2 | GAGAAAAAAAAATACATGCGTAAGTACATTAAAATTACAGGAAAAATCTTAATCGATGAATTCGAGCTCG | *PAT1* deletion |
| PAT1-S3 | CTAAACGTTATGGGGTTGGTGTATCGCGATGGTGAAATATCAGAACTAAAGCGTACGCTGCAGGTCGAC | *PAT1* deletion |
| ASH1-MS2F | CTTATTTTGTAATTACATAACTGAGACAGTAGAGAATTGAACGCTGCAGGTCGACAACCC | *ASH1-MS2L* tagging |
| ASH1-MS2R | ATGTCTCTTATTAGTTGAAAGAGATTCAGTTATCCATGTAGCATAGGCCACTAGTGGATC | *ASH1-MS2L* tagging |
| ASH1-F | CTGCGAAATTGAAGGGTACCG | *ASH1-MS2L* checking |
| ASH1-R | GCACAGACAAGGAGAGAAATG | *ASH1-MS2L* checking |
| GPD1-MS2F | CCCACTTTTTTCGAGGCTCTTCTATATCATATTCATAAACGCTGCAGGTCGACAACCC | *GPD1-MS2L* tagging |
| GPD1-MS2R | AACGTGATAAAGTAGTTATGAGAAATGACATAATGCTAATGCATAGGCCACTAGTGGATC | *GPD1-MS2L* tagging |
| GPD1-F | CAACTACCCAATGAAGAACC | *GPD1-MS2L* checking |
| GPD1-R | GAAGAGCCTCGAAAAAAGTG | *GPD1-MS2L* checking |
| GPD1-ORF-F | GATCTGGTAACTGGGGTACT | *GPD1-MS2L* checking |
| GPD1-ORF-R | ATGATCTCACCCAAACCGAC | *GPD1-MS2L* checking |
| STL1-MS2F | TTGCGGGGTTTATATTTGTTCCTTGCCGCACCAATAATATACGCTGCAGGTCGACAACCC | *STL1-MS2L* tagging |
| STL1-MS2R | GTCTGGTGTTAAAATACTAAAGTTATGTAGTAATATATGAGCATAGGCCACTAGTGGATC | *STL1-MS2L* tagging |
| STL1-F | CGTCTAGTTCTTCAAACATC | *STL1-MS2L* checking |
| STL1-R | CATATTTCCTCAAAAGACAG | *STL1-MS2L* checking |
| STL1-ORF-F | GCCGTTATTTCTACATGCGC | *STL1-MS2L* checking |
| STL1-ORF-R | GTAGATTGTTGCGAAGACCC | *STL1-MS2L* checking |
| HYP2-MS2F | AGCCGCCATCTCCTTCAAGGAAGCTGCTAGAACCGATTAAACGCTGCAGGTCGACAACCC | *HYP2-MS2L* tagging |
| HYP2-MS2R | TCTTTTTTCATTTATATCCCATGCCATGATGTTAACCGGTGCATAGGCCACTAGTGGATC | *HYP2-MS2L* tagging |
| HYP2-F | CATCATCTCC GCTATGGGTG | *HYP2-MS2L* checking |
| HYP2-R | GTGATGGAAGGGGCGTCGGAG | *HYP2-MS2L* checking |
| ACT1-qPCR-F | TCGTTCCAATTTACGCTGGTT | qPCR |
| ACT1-qPCR-R | CGGCCAAATCGATTCTCAA | qPCR |
| GPD1-qPCR-F | GCGCTGGTGGTAGAAACGTCA | qPCR |
| GPD1-qPCR-R | TCCCAGGCGTCCTTACCAGA | qPCR |
| STL1-qPCR-F | CCGGAAGAAGTTTGGAGGAA | qPCR |
| STL1-qPCR-R | GGGCAAATGGTTAGCAACTC | qPCR |
| ASH1-qPCR-F | TCCACACCGACGAAAAGTGG | qPCR |
| ASH1-qPCR-R | TGTATGCCTTGGGACGCACA | qPCR |
| HYP2-qPCR-F | TTGATGAACATGGACGGTGACA | qPCR |
| HYP2-qPCR-R | TGTCACCCAATTCACCTTCTGG | qPCR |
| PWP1-qPCR-F | TACGAACACTAAGGGCCCAAGC | qPCR |
| PWP1-qPCR-R | TCCGACATCGAAATCACGAGAC | qPCR |
| UTP13-qPCR-F | GAGCTTTGGGCGAATCAAGG | qPCR |
| UTP13-qPCR-R | GACTTCAATTTTGCCCTCTTCG | qPCR |
| GRE3-qPCR-F | CAAAACCATCCAGGCAGTAC | qPCR |
| GRE3-qPCR-R | CTCTCTGAGTTGCCCATCTA | qPCR |
| GPP2-qPCR-F | TGCAACGCTTTGAACGCTCT | qPCR |
| GPP2-qPCR-R | GATTCCCAGATGCTCGAACCA | qPCR |
| LSB1-qPCR-F | TCATACGAACCAGCCGCATC | qPCR |
| LSB1-qPCR-R | AACCGGCTGGAGGTGCATAA | qPCR |
| QCR6-qPCR-F | CAGCAACAACAACCCGGCTA | qPCR |
| QCR6-qPCR-R | GCCGTGGCAGTGTCCAAATA | qPCR |
| CWP2-qPCR-F | ACCACTGCTACCACCGAAGCTA | qPCR |
| CWP2-qPCR-R | CGGTGCTGGATGGAGAAACA | qPCR |
| TMA10-qPCR-F | GCAAGCCTGGCGATGAGATT | qPCR |
| TMA10-qPCR-R | GAGCCCCTTCTGGTCTTGTTGA | qPCR |
| FBA1-qPCR-F | GCTTTGCACCCAATCTCTCCA | qPCR |
| FBA1-qPCR-R | TCACCAGCGTACAAACCGTGA | qPCR |
| ENO1-qPCR-F | CTGCCGACGCTTTGTTGTTG | qPCR |
| ENO1-qPCR-R | CGGCAGCGAAAGAGTCTTGA | qPCR |
| HAC1-qPCR-F | CAGTTCGCACTCGTCGTCTGAT | qPCR |
| HAC1-qPCR-R | CGCATACTCTTGGGCGACAA | qPCR |
